# Iron-dependent mutualism between *Chlorella sorokiniana* and *Ralstonia pickettii* forms the basis for a sustainable bioremediation system

**DOI:** 10.1038/s43705-022-00161-0

**Published:** 2022-09-15

**Authors:** Deepak Rawat, Udita Sharma, Pankaj Poria, Arran Finlan, Brenda Parker, Radhey Shyam Sharma, Vandana Mishra

**Affiliations:** 1 Bioresources & Environmental Biotechnology Laboratory, Department of Environmental Studies, University of Delhi, Delhi, 110007 India; 2 Department of Biochemical Engineering, Bernard Katz Building, University College London, Gower Street, London, WC1E 6BT UK; 3 Department of Environmental Studies, Janki Devi Memorial College, University of Delhi, Delhi, 110060 India; 4 Delhi School of Climate Change & Sustainability, Institute of Eminence, University of Delhi, Delhi, 110007 India; 5 Centre for Interdisciplinary Studies on Mountain & Hill Environment, University of Delhi, Delhi, 110007 India

**Keywords:** Environmental sciences, Microbial ecology

## Abstract

Phototrophic communities of autotrophic microalgae and heterotrophic bacteria perform complex tasks of nutrient acquisition and tackling environmental stress but remain underexplored as a basis for the bioremediation of emerging pollutants. In industrial monoculture designs, poor iron uptake by microalgae limits their productivity and biotechnological efficacy. Iron supplementation is expensive and ineffective because iron remains insoluble in an aqueous medium and is biologically unavailable. However, microalgae develop complex interkingdom associations with siderophore-producing bacteria that help solubilize iron and increase its bioavailability. Using dye degradation as a model, we combined environmental isolations and synthetic ecology as a workflow to design a simplified microbial community based on iron and carbon exchange. We established a mutualism between the previously non-associated alga *Chlorella sorokiniana* and siderophore-producing bacterium *Ralstonia pickettii*. Siderophore-mediated increase in iron bioavailability alleviated Fe stress for algae and increased the reductive iron uptake mechanism and bioremediation potential. In exchange, *C. sorokiniana* produced galactose, glucose, and mannose as major extracellular monosaccharides, supporting bacterial growth. We propose that extracellular iron reduction by ferrireductase is crucial for azoreductase-mediated dye degradation in microalgae. These results demonstrate that iron bioavailability, often overlooked in cultivation, governs microalgal growth, enzymatic processes, and bioremediation potential. Our results suggest that phototrophic communities with an active association for iron and carbon exchange have the potential to overcome challenges associated with micronutrient availability, while scaling up bioremediation designs.

## Introduction

Phototrophs and heterotrophs occupy distinct ecological niches by developing mutualism to complement their physiological capabilities and metabolic versatilities [1, 2]. Such phototrophic communities of co-evolving microorganisms perform complex tasks of nutrient acquisition and combat environmental stress [3–6]. In aquatic ecosystems, photoautotrophic algae exchange dissolved organic matter (DOM) with heterotrophic bacteria in lieu of biologically unavailable micronutrients like iron and vitamins to maintain fitness [7, 8]. However, the role of mutualism in improving the sustainability of biotechnological processes is largely unexplored [9–11]. Complex environmental microbial communities are difficult to isolate, replicate, and study for industrial applications in biotechnological processes. Therefore, microbial designs, especially in bioremediation, are mostly restricted to single-species/ monocultures [9, 12], which focus on the biodegradation of contaminants under controlled laboratory conditions. However, single-species designs are limited to a handful of known mechanistic/enzymatic processes that pose physiological constraints for bioremediation applications at the industrial scale.

Synthetic dyes arising from textile industries account for 1/5th of global water pollution [13] and constitute an emerging threat to the environment and human health [14–17]. Microbial bioremediation of dyes has shown potential but faces many challenges in their translation from laboratories to industrial scale. The bacterial remediation approaches are difficult to integrate into textile industries as they require varied oxygen conditions for dye degradation and nutrient supplementation for heterotrophic growth [18, 19]. In comparison, microalgae-based industrial remediation systems are promising due to the rapid growth of autotrophic organisms, high catabolic diversity, diverse enzymatic machinery, oxygen regulation, and high adaptability to low nutrient conditions [20, 21]. Several microalgae, such as *Chlamydomonas*, *Chlorella*, *Scenedesmus*, and *Phaeodactylum*, have been investigated for wastewater treatment [22–27], including the remediation of synthetic dyes [28, 29]. However, algal monocultures face several physiological constraints, especially in polluted environments, such as low bioavailability of micronutrients (Fe, Mn, vitamins) and a high metabolic burden to maintain essential cellular processes [9, 30, 31]. Microalgae require ferrous (Fe^2+^) ions for photosynthesis, respiration, nitrogen fixation, and the metabolism of reactive oxygen species. However, algae lack the ability to secrete chelators that can solubilize inorganic iron to form bioavailable iron complexes [32], thus, they require supplements of external chelators like EDTA to increase iron bioavailability. Although abundant in the environment, iron remains unavailable to algae due to its presence as insoluble ferric (Fe^3+^) or oxyhydroxides complexes and precipitation in alkaline pH [33, 34], which is also a characteristic feature of textile wastewater [35]. Therefore, iron is a limiting nutrient for microalgal growth in industrial wastewater and must be externally supplied for optimum cell growth.

Employing mutualistic microalgae and bacteria in co-culture may serve as a sustainable bioremediation option, allowing the culture members to share the metabolic burden and provide complementary physiological capabilities for resource capture [4]. In iron-stressed aquatic ecosystems, some heterotrophic bacteria produce siderophores, i.e., low molecular weight iron chelators, as a part of high-affinity iron-uptake mechanisms [1], promoting mutualistic associations with microalgae [8, 36]. Siderophore-mediated iron chelation increases iron pools for quick uptake by algae via plasma membrane-bound ferrireductase enzyme [37], and in return, algae provide carbon for bacterial growth. Therefore, employing mutualistic communities in bioremediation can help overcome iron and carbon limitation that otherwise restricts the application of monocultures of algae and bacteria [9]. Designing synthetic communities, which are simpler representations of a complex environment, enabling us to test theories of microbial ecology [38], and enhance our understanding of microbial interactions [39]. These models can be further extended to bioremediation to address the challenges of current monoculture designs by offering a platform to develop and test numerous hypotheses [40], find novel enzymes and degradation pathways [9], and integrate ecological principles of mutualism [41].

Using dye degradation as a model, we combined environmental isolation and synthetic ecology workflows to generate a simplified self-sustainable phototrophic community with microalga and siderophore-producing bacteria. We isolated the siderophore-producer bacteria *Ralstonia pickettii* from industrial wastewater and demonstrated its ability to undergo mutualism with the dye degrader microalga *Chlorella sorokiniana* to overcome the challenges of iron and carbon availability in bioremediation. In this study, siderophore-producing bacteria enhanced reductive iron uptake and bioremediation potential of freshwater microalgae under iron-limiting conditions, demonstrating the role of siderophore-producing bacteria in enhancing algal growth by alleviating Fe stress.

## Results and discussion

### Iron and carbon dependent mutualism between *Chlorella sorokiniana* and *Ralstonia pickettii* forms a synthetic phototrophic community

**Fig. 1:**
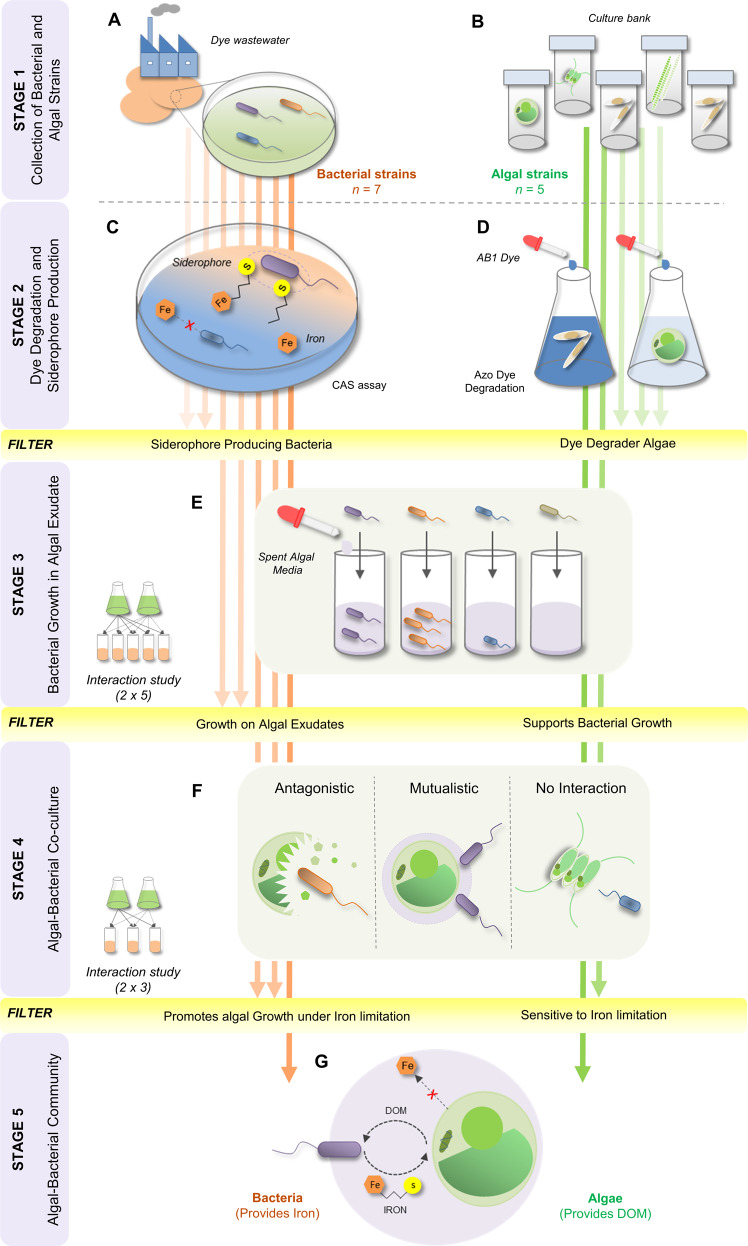
The study design explains different stages of experiments to develop a phototrophic community of previously non-associated algae and bacteria. The stages include (**A**) isolation of bacterial strains from textile wastewater collected from Panipat Industrial area, Haryana (India); **B** cultivation of freshwater and marine algal strains; **C** assessment of siderophore production in bacterial strains using Schwyn and Neilands’s universal Chrome Azurol S (CAS) assay; **D** assessment of dye degradation potential of algae strains using Acid Black 1 (AB1) dye; **E** interaction study between siderophore producing bacteria and dye degrader microalgae to identify bacterial strains that could sustain on algae-derived DOM secreted in algal exudates; **F** algal-bacterial co-culturability assessment to study different types of microbial interactions viz. antagonism, mutualism, or no interaction between the two organisms, and **G** identification of algal-bacterial model phototrophic community based on the active exchange of iron and DOM (refer to Data S1 for detailed results).

The synthetic microalgal-bacterial community based on the active exchange of iron and carbon was developed by screening multiple siderophore producer bacteria and dye decolorizer algae (Fig. 1; refer to Supplementary Data S1 for detailed results). Out of seven bacterial isolates obtained from untreated textile wastewater, five showed relatively high siderophore production in CAS agar plates and broth (Fig. S1). In broth, *Serratia plymuthica* PW1, *Serratia liquefaciens* PW71, and *Ralstonia pickettii* PW2 produced siderophores in decreasing order of concentration, i.e., 15.26 ± 1.3 > 13.28 ± 0.9 > 10.85 ± 0.7 µMmL^−1^ (Table 1). Arnow’s assay confirmed that *S. plymuthica* PW1 (81.10 ± 9.8 µMmL^−1^), *R. pickettii* PW2 (97.43 ± 16.8 µMmL^−1^), and *S. liquefaciens* PW71 (103.1 ± 8.3 µMmL^−1^) produced catecholate-type siderophores. On the other hand, Csaky’s assay confirmed that *Stenotrophomonas maltophilia* PW5 (37.86 ± 0.4 µMmL^−1^) and *Stenotrophomonas maltophilia* PW6 (17.73 ± 0.2 µMmL^−1^) produced hydroxamate-type of siderophores. Out of the five algal species, only freshwater microalgae *Chlorella sorokiniana* and *Scenedesmus* sp. showed the highest dye degradation potential; therefore, they were selected for further experiments (Data S1).

**Table 1:** Characterization of siderophore production in bacterial strains isolated from textile wastewater.

Bacteria	Concentration (µmol mL^−1^)		
	Universal - CAS assay (w.r.t. Desferrioxamine mesylate)	Catecholate - Arnow’s assay (w.r.t. 2’3-Dihydroxybenzoic acid)	Hydroxamate - Csaky’s assay (w.r.t. Desferrioxamine mesylate)
*Serratia plymuthica* PW1	15.26 ± 1.28	81.10 ± 9.85	1.35 ± 1.03
*Ralstonia pickettii* PW2	10.85 ± 0.70	97.43 ± 16.86	NA
*Stenotrophomonas rhizophila* PW3	1.31 ± 0.40	NA	NA
*Stenotrophomonas maltophilia* PW5	1.28 ± 0.34	2.77 ± 1.20	37.86 ± 0.46
*Stenotrophomonas maltophilia* PW6	1.63 ± 0.07	2.43 ± 0.33	17.73 ± 0.26
*Serratia liquefaciens* PW71	13.28 ± 0.92	103.1 ± 8.33	NA
*Stenotrophomonas rhizophila* PW72	0.91 ± 0.63	7.4 ± 6.86	NA

After that, the sterile exudates from *C. sorokiniana* and *Scenedesmus* sp. were used as the sole source of dissolved organic matter for bacterial growth and selection of appropriate microalgal-bacterial partners comprising the phototrophic community (Fig. 1E; Data S2). All five bacterial isolates grew well on the exudate of *C. sorokiniana* as a sole source of carbon. On the contrary, on exudates of *Scenedesmus* sp., *S. plymuthica* PW1 showed moderate growth in 20 h, while the growth of *R. pickettii* PW2 and *S. liquefaciens* PW71 remained insignificant. S. maltophilia PW5 and PW6 failed to grow in the exudate of *Scenedesmus* sp. (Fig. S2B).

**Fig. 2:**
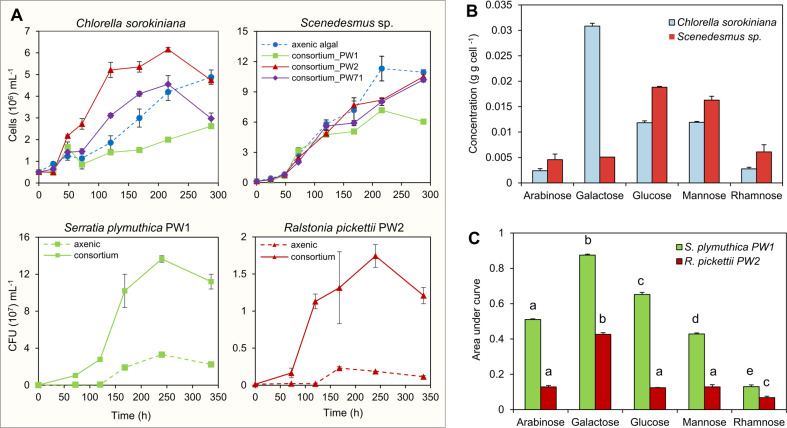
Assessment of algal and bacterial growth in co-culture experiments. **A** The growth curves represent the difference in the growth of *C. sorokiniana* when grown axenically or in co-culture with *S. plymuthica* PW1, *R. pickettii* PW2, and *S. liquefaciens* PW71 under iron limiting conditions. Whereas, the effect of bacteria on the growth of *Scenedesmus* sp. was less prominent. The difference in the CFUs of bacterial strains in axenic culture and co-culture suggests the growth-promoting effect of *C. sorokiniana* on *S. plymuthica* PW1 and *R. pickettii* PW2. **B** Anion-exchange chromatography suggests a difference in the glycosyl composition in the EPS of *C. sorokiniana* and *Scenedesmus* sp. **C** The area under curve (auc) of *S. plymuthica* PW1 and *R. pickettii* PW2 obtained after growth curves in different sugars. Here, ‘a’, ‘b’, etc., represent grouping after Tukey’s post hoc test.

**Fig. 3:**
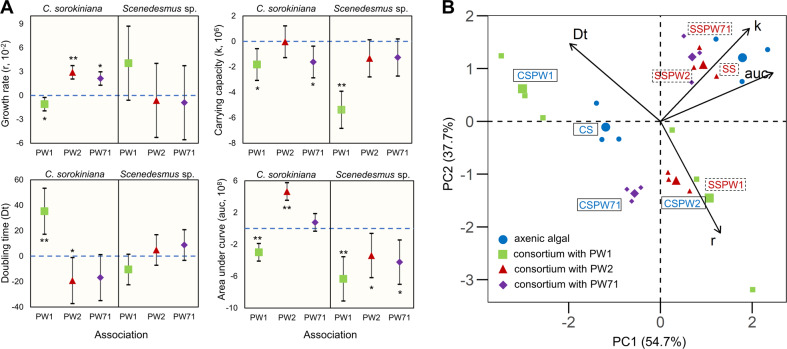
Assessment of algal growth parameters in the algal-bacterial phototrophic community under iron-limiting conditions. **A** The confidence interval plots represent the significant difference in the growth parameters i.e., growth rate ‘r’, carrying capacity ‘k’, doubling time ‘Dt’, and area under curve ‘auc’, of *C. sorokiniana* (left panel) and *Scenedesmus* sp. (right panel) in algal-bacterial co-cultures w.r.t. to axenic culture (horizontal blue dashed line). The symbols ‘*’ and ‘**’ represent *p* values with statistical significance of ‘*p* < 0.05’ and ‘*p* < 0.001’ res*p*ectively. **B** The PCA biplot represents the separation of axenically grown algae *C. sorokiniana* (CS) and *Scenedesmus* sp.(SS) from algae grown in co*-*culture with *S. plymuthica* PW1 (CSPW1/ SSPW1), *R. pickettii* PW2 (CSPW2*/* SSPW2), and *S. liquefaciens* PW71 (C*S*PW71/SSPW71) based on different growth parameters (refer to Data S2 for detailed results).

Finally, the compatibility between the phototrophic community of selected microalgae (*C. sorokiniana*/ *Scenedesmus* sp.) and siderophore-producer bacteria (*S. plymuthica* PW1/ *R. pickettii* PW2/ *S. liquefaciens* PW71) was tested by co-culturing them in iron limiting BBM media (BBM-Fe; without EDTA) (Fig. 1F). In the absence of EDTA, Fe precipitates rapidly as iron oxyhydroxides and becomes unavailable to microbes. Microalgal growth curves in co-culture assays were used to measure and compare population characteristics such as carrying capacity ‘k’, growth rate ‘r’, etc., in axenic and consortium setups. Algal growth parameters in co-culture with a bacterial partner were used to categorize their interaction as putative mutualistic, antagonistic, and neutral (Data S1, Tables S1 and S2) [42]. Under iron-limiting conditions, axenic *C. sorokiniana* experienced iron stress as the cell growth was 4.2 ± 0.4 × 10^6^ cells mL^−1^ after 200 h incubation. On the other hand, axenic *Scenedesmus* sp. showed a significantly higher growth (11.3 ± 1.2 × 10^6^ cells mL^−1^) than *C. sorokiniana* suggesting an effective iron uptake mechanism under iron-limiting conditions (k; *t*-test, *p* = 0.001) (Table S1). In contrast to the axenic microalgal culture, *C. sorokiniana* in co-culture with *R. pickettii* PW2 showed a significant increase in cell count at 200 h (6.2 ± 0.85 × 10^6^ cells mL^−1^) (auc; *p* = 0.000). However, *S. plymuthica* PW1 exerted a negative effect on *C. sorokiniana* (Fig. 2A), as indicated by its significant increase in doubling time (*p* = 0.009) and reduction in auc (*p* = 0.001) (Fig. 3A). While *S. liquefaciens* PW71 remained neutral to *C. sorokiniana* (auc; *p* = 0.430) (Fig. 2A, Table 2). On the other hand, the interaction of *Scenedesmus* sp. with both *R. pickettii* PW2 and *S. liquefaciens* PW71 was neutral, while *S. plymuthica* PW1 showed a negative effect (Figs. 2A and 3A).

As indicated by a steeper slope of the log-phase in the growth curve (Fig. 3A), the growth rate ‘r’ of *C. sorokiniana* in the consortium with *R. pickettii* PW2 (5.02 ± 1.0 × 10^−2^ h^−1^) remained significantly higher than that of axenically grown microalgae (1.95 ± 0.3 × 10^−2^ h^−1^) (*p* = 0.000). In the consortium, *C. sorokiniana* showed a higher population turnover during the early log-phase and reached the stationary phase earlier (at 100 h) than cells grown axenically (~270 h), although the carrying capacity remained similar (Fig. 3A), suggesting that algae grew faster under iron-limiting conditions in the consortium. In addition, the NO_3_-N drawdown in media under axenic and consortium setups was monitored to assess whether the difference in N-uptake led to an increase in algal growth in consortium setup. After 310 h, the nitrate concentration dropped from 123 to 74.75 ± 3.15 and 80.25 ± 1.85 mgL^−1^ in axenic and consortium setups, respectively, suggesting only a slight difference between the two setups. The higher growth of *C. sorokiniana* in consortium setup was not because of the difference in N-uptake, but because of the difference in iron bioavailability (Fig. S3C). Thus, iron was the only growth-limiting factor in the BBM. The lower bioavailability of iron because of the absence of chelating agents like EDTA in iron-deficient BBM reduced algal growth. However, the presence of siderophore producer *R. pickettii* in co-culture increased the growth rate of *C. sorokiniana* under iron-limiting conditions [43].

The principal component analyses (PCA) biplot further explained the difference in the growth of *C. sorokiniana* and *Scenedesmus* sp. in axenic and co-culture setups (Fig. 3B; refer to Supplementary Data S2 for detailed analysis). Grouping of *Scenedesmus* sp. grown axenically (SS), in consortium with *R. pickettii* PW2 (SSPW2) and *S. liquefaciens* PW71 (SSPW71) due to similar area under curve ‘auc’ and carrying capacity ‘k’ parameters, suggested a neutral interaction. Both ‘auc’ and ‘k’ contributed to PC1 by 39.17% and 24.39%, respectively (Fig. S2D). The separation based on higher ‘auc’ of *Scenedesmus* than *Chlorella* was due to their different growth responses under iron limitation (Fig. 2A), which might govern their interactions with bacteria (Fig. 3A). In contrast with *Scenedesmus*, *C. sorokiniana* grown in consortium with *R. pickettii* PW2 (CSPW2) was separated from axenically grown algae (CS) based on higher growth rate ‘r’ (Fig. 3B). PC2 explains 37.7% of the grouping of different variables, which had ‘r’ as the dominant metric (42.25%). On the contrary, CSPW1 co-culture was separated from axenic *C. sorokiniana* due to a higher doubling time ‘Dt’, indicating a negative effect of *S. plymuthica* on the growth of *Chlorella* (Fig. 2A).

Such an iron-dependent mutualism has been previously reported between alga *Dunaliella bardawil* and *Halomonas* sp [44]., diatom *Navicula pelliculosa* and *Cupriavidus necator* [45], marine alga *Scrippsiella trochoidea* and *Marinobacter* sp [37]., freshwater alga *Chlorella variabilis* and *Idiomarina loihiensis* [46]. Previously, a commensal association between *R. pickettii* and *C. sorokiniana* has been reported under nutrient-sufficient photoautotrophic conditions [47]. In this study, compared with axenic culture, *R. pickettii* PW2 showed a higher growth when supplemented with exudate of *Chlorella* than *Scenedesmus*, indicating the use of *Chlorella*-derived organic matter as a preferred substrate for growth (Fig. S2B). *R. pickettii* PW2 also showed enhanced growth in co-culture with *Chlorella* compared to axenic culture (Fig. 2A), which suggested a mutualistic association between algae and bacteria.

Algal exopolysaccharides (EPS) serve as a carbon source for bacteria and influence microbial interactions [48, 49]. The HPAEC analyses of EPS of *C. sorokiniana* detected commonly reported galactose (0.03 ± 0.0 g gcell^−1^; 52%, relative percentage) as the dominant monosaccharide besides glucose (20%), mannose (20%), arabinose (4%), and rhamnose (4%). The EPS of *Scenedesmus* sp. had glucose (0.31 ± 0.0 g gcell^−1^, 37%) as the dominant monosaccharide, followed by mannose (32%), rhamnose (12%), galactose (10%), and arabinose (9%) (Figs. 2B and S3A). In a bacterial growth assay performed on the 5 monosaccharides, mutualistic bacterium *R. pickettii* PW2 showed significantly higher growth in galactose (auc; *p* = 0.001) (Fig. 2B, C and Table S15). The higher presence of galactose in EPS has been hypothesized to have a role in maintaining an extended stationary phase in green algae [50]. The putative antagonistic *S. plymuthica* PW1 showed ~10 times higher growth than *R. pickettii* PW2 when grown with *C. sorokiniana* (Fig. 2A). *S. plymuthica* PW1 also grew well on supplementing with any of the five monosaccharides, with galactose being the preferred carbon source (Fig. 2C). Thus, the negative effect of *S. plymuthica* PW1 on *C. sorokiniana* could have been due to its aggressive growth and generalist behaviors. Therefore, the composition of algal EPS and its metabolism by bacteria could have influenced the nature of algal-bacterial interactions [51, 52]. However, further studies at the molecular level will ascertain the influence of EPS composition to initiate and maintain such associations. Apart from the tested monosaccharides, algae produce several organic compounds that could also act as a substrate and influence such associations [53]. Thus, our study posits that *Chlorella* EPS can serve as a source of DOM for *R. pickettii* PW2 to form a mutualistic association in exchange for bioavailable iron. Consequently, these algal-bacterial partners were selected to form the phototrophic community.

### Bioavailable iron influences dye degradation of phototrophic community

**Fig. 4:**
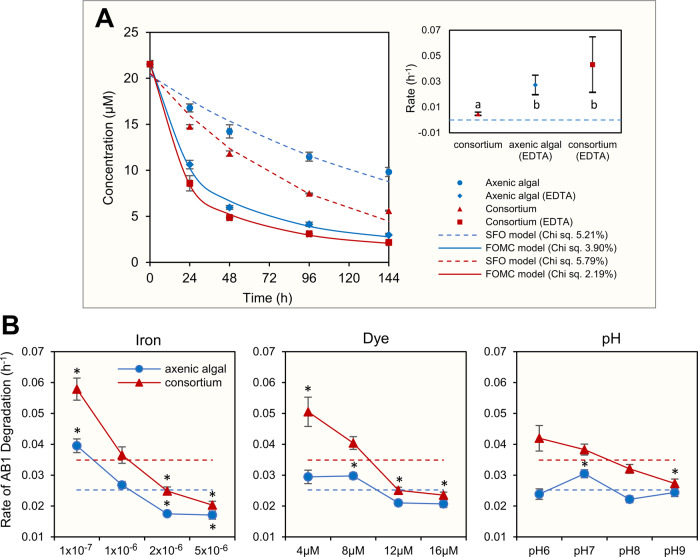
Dye degradation assessment of the algal-bacterial phototrophic community. **A** Degradation of Acid Black 1 (AB1) dye followed a Single First Order (SFO) kinetics without EDTA-chelated iron and bi-phasic First Order Multi-compartment (FOMC) kinetics with EDTA-chelated iron. The confidence interval plot represents a significant difference between the rate of AB1 degradation in axenic culture (with and without EDTA chelated iron) and consortium culture (with and without EDTA chelated iron). The Blue dashed line represents axenic alga grown without EDTA-iron. Here, ‘a’ and ‘b’ represents grouping after Tukey’s post hoc test. **B** The output of Taguchi’s orthogonal array represents the impact of change in iron concentration majorly affecting AB1 degradation rate, followed by the change in dye concentration and pH (refer to Table S7 for details).

To ascertain the significance of biologically available iron in bioremediation, the dye degradation potential of *C. sorokiniana* was analyzed in axenic and consortium cultures in iron-deficient (without EDTA) and -sufficient (with EDTA) conditions. In axenic culture, dye degradation rate was 0.005 ± 0.000 h^−1^ under iron-deficient conditions, which increased to 0.033 ± 0.003 h^−1^ when iron was more bioavailable (Fig. 4A). In contrast, the dye degradation rate was higher in consortium setups under iron-deficient conditions (0.009 ± 0.000 h^−1^), which increased to 0.049 ± 0.008 h^−1^, when iron was more bioavailable.

Under iron-deficient conditions, the difference in the dye degradation rate between axenic and consortium setups was significant (*p* = 0.000) (Tables S3 and S4). The presence of siderophore-producing *R. pickettii* significantly increased the dye degradation potential of *C. sorokiniana*, whereas, the axenic bacteria lacked detectable dye degradation suggesting a significant contribution of a microalgal partner in dye degradation in the consortium. Although, in iron-sufficient conditions, the dye decolorization by *C. sorokiniana* in both axenic alga and consortium increased, but the difference between them was not statistically significant (*p* = 0.126) (Fig. 4A).

In the iron-deficient conditions, the dye degradation followed Simple First Order (SFO) kinetics (χ^2^ value, axenic alga: 6.02%; consortium: 8.56%), as also reported in other microbial dye degradation studies (Table S3) [54–56]. In SFO kinetics, the rate of degradation depends on the concentration of reactant. In contrast, dye degradation followed a First Order Multi-Compartment (FOMC) kinetics in iron-sufficient conditions, which is characterized by a biphasic degradation indicating an initial steep decline in dye concentration followed by a relatively slower degradation (χ^2^ value, axenic alga: 4.20%; consortium: 2.86%) (Fig. 4A). *C. sorokiniana* in co-culture with *R. pickettii* showed a significantly high dye degradation only in the iron-deficient condition (Fig. 4A). Under iron-deficient conditions, bacteria produce siderophores to chelate iron and make it bioavailable to both bacteria and algae [57]; however, because EDTA is a strong chelator, bioavailable iron significantly increased (Fig. 4A and Table S4). Thus, in iron-sufficient conditions, bacterial presence lacks any significant effect on algal dye degradation due to the high bioavailability of iron because of EDTA (*p* = 0.126). Thus, *R. pickettii* PW2 increased the dye degradation potential of *C. sorokiniana* only under iron-limiting conditions.

L_16_ (4^3^) design was used to compare the effect of varying Fe^3+^, dye concentration, and pH level on microalgal dye remediation potential in axenic and consortium setups (Fig. S5, Table S5). The Fe^3+^ concentration was kept lower or higher than 1 × 10^−6 ^M, a concentration known to induce iron-starvation due to variation in the equilibrium between intra- and extracellular iron, thus, necessitating bacterial siderophore production [58, 59]. Iron precipitation increases with increasing pH, reducing its solubility and bioavailability [60]. Since the pH of the textile wastewater varies from 6 to 10, the effect of varying pH on dye degradation was tested [35]. The effect of varying AB1 dye (substrate) concentration was tested because it affects the enzymatic activity that determines the rate of dye degradation.

Results of the multiple regression model suggested Fe^3+^ concentration (delta value; axenic alga = 0.02, consortium = 0.03) as a primary factor governing the rate of dye degradation, followed by the azo dye concentration (delta value; axenic alga = 0.01, consortium = 0.02) (Fig. 4B, Tables S6 and S7). The delta value in Taguchi’s orthogonal design takes all the factors individually to determine the difference between the highest and lowest values of the average response variable. Therefore, a higher delta value of a particular factor represents a significant effect of variation in the level of the factor. Changing the concentration of Fe^3+^ led to a considerable variation in the rate of AB1 degradation in both axenic algal (*p* = 0.001) and consortium (*p* = 0.002) setups (Fig. 4B). Further analysis using Partial Least Squares Path (PLSP) modeling suggested that the rate of AB1 degradation was inversely proportional to Fe concentration; the effect was prominent in consortium setup (Fig. S4B, Table S16). The consortium setup showed an enhanced average rate of AB1 degradation (0.04 h^−1^) as compared to axenic cultures (0.03 h^−1^) (Fig. 4B). Siderophore-producer bacteria increased the dye degradation potential of microalga at 1 × 10^−7^ and 1 × 10^−6 ^M Fe, whereas at a higher Fe^3+^ concentration, the bacterial effect remained neutral.

The dye concentration also showed an inverse relationship with the rate of dye degradation in both axenic algal and consortium setups, but the effect was prominent in consortium setup (Fig. 4B, Table S7), as suggested by PLSP analysis (Fig. S4B, C, Table S16). Although the rate of dye degradation decreased with increasing dye concentration, the algal-bacterial consortium could degrade up to ~60% dye even at higher dye concentrations (Fig. 4B, Table S5). At a high concentration, the dye molecules compete for electrons generated by the azoreductase-mediated enzymatic mechanism at the microbial membrane [61], thus reducing the dye degradation rate. Similarly, the siderophore-producer bacteria only increased the dye degradation rate at low dye concentrations (Fig. 4B, Tables S6 and S7). Dye degradation in bacteria is a non-growth associated extracellular process driven by membrane-bound azoreductase, a highly diverse and non-specific oxidoreductase. Azoreductases have been widely reported in bacteria, such as *P. aeruginosa*, *E. coli*, and *Bacillus* sp [62, 63]., but not from algae. Bacterial azoreductase facilitates electron transfer from cells to electron-deficient azo bond (-N = N-), which reduces azo dyes into colorless aromatic amines via a two-cycle transfer of electrons following a ping-pong bi-bi mechanism [18, 61]. Azoreductases use extracellular mediators such as flavins which transfer electrons from within the microbial cell to outside for azo dye reduction. Therefore, the optimal microalgal cell-to-dye ratio significantly influences catalytic efficiency and enzyme turnover for azo dye degradation. On the contrary, pH lacked any significant effect (*p* > 0.1) on rates of AB1 degradation in both consortium and axenic algal setups (delta value; axenic alga = 0.01, consortium = 0.01), although, the rate of degradation was higher at the lower pH in the consortium setup (Fig. 4B, Table S6). The least impact of the varying pH on the rate of AB1 degradation could also have been due to pH regulation of the culture media by algal photosynthesis and N uptake. *C. sorokiniana* changed the pH of the neutral BBM to 7.9, suggesting a pH regulation due to algal photosynthesis. This increase in pH has been linked to the consumption of CO_2_ and the nitrate metabolism in closed and stationery phototrophic culture conditions [64].

The PLSP model revealed the positive effect of interactions between factors Fe*pH and Fe*Dye on the rate of AB1 degradation in the consortium setup (Fig. S4B). The results suggest that variation in Fe positively influenced the effect pH and Dye concentration had on the rate of dye degradation. However, the interaction effect was significant only in consortium setups, thus, suggesting the presence of bacteria plays a key role in ensuring Fe availability for algae and also the dye degradation potential. However, further analysis of the interactions between abiotic factors in a full factorial design will help determine the influence of factors on each other.

### Plasma membrane-bound ferrireductase enzyme influences algal dye degradation

**Fig. 5:**
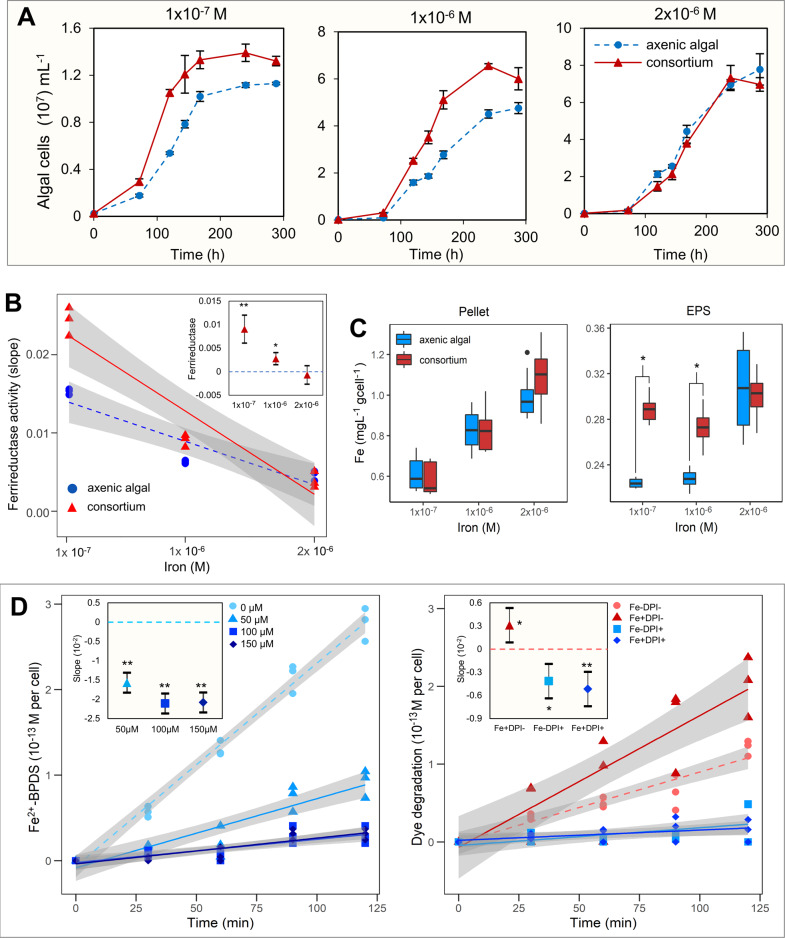
Impact of iron concentration on the growth and enzyme activity of *C. sorokiniana*. **A** Algal growth curve represents the difference in the growth of *C. sorokiniana* in axenic and consortium cultures in different iron concentrations. **B** The difference in ferrireductase enzyme activity of *C. sorokiniana* in axenic culture and algal-bacterial consortium under different iron concentrations (inset; confidence interval plot representing the statistical significance of the difference). **C** The ICP-MS analysis of iron concentration in cell pellet and EPS under different iron concentrations of *C. sorokiniana* grown axenically or in the consortium. **D** The difference in the ferrireductase and azoreductase enzyme activity of *C. sorokiniana* at different Diphenyleiodonium (DPI) concentrations and treatment conditions of Iron (Fe) and DPI (50 µM). Here, Fe-DPI-, Fe+DPI-, Fe-DPI + , and Fe+DPI + represent different treatment setups and ‘+’ and ‘-’ denote presence and absence. The symbols ‘*’ and ‘**’ represent *p* values with statistical significance of ‘*p* < 0.05’ and ‘*p* < 0.001’ res*p*ectively.

We hypothesize that the increased rate of azo dye degradation with increased bioavailable iron was associated with higher azoreductase activity per cell or was due to increased algal cell numbers. Therefore, the effect of varying iron concentrations on the algal cell growth, enzymatic activities, and iron uptake was investigated in axenic algal and algal-bacterial consortium setups. With the increase in iron concentration, the algal cell growth was increased in both axenic and consortium setups; however, the difference in algal growth in the two setups was significant only in lower iron concentrations (Fig. 5A). In axenic cultures, *C. sorokiniana* growth varied with Fe concentration in experimental setups, 2 × 10^−6 ^M Fe: 8 × 10^7^ cells mL^−1^, 1 × 10^−6 ^M Fe: 6 × 10^7^ cells mL^−1^, and 1 × 10^−7 ^M Fe: 1.4  × 10^7^ cells mL^−1^. At lower Fe concentrations, *R. pickettii* PW2 enhanced the growth of *C. sorokiniana*. The positive effect of the siderophore-producing bacteria on algal growth was also significant at lower Fe concentrations, including growth parameters, such as growth rate ‘r’: *p* = 0.006 for 1 × 10^−7 ^M Fe, *p* = 0.028 for 1 × 10^−6 ^M Fe; carrying capacity ‘k’: *p* = 0.025 for 1 × 10^−7 ^M Fe, and *p* = 0.017 for 1 × 10^−6 ^M Fe; and area under curve ‘auc’: *p* = 0.003 for 1 × 10^−7 ^M Fe, and *p* = 0.001 for 1 × 10^−6 ^M Fe (Fig. S6, Tables S8 and S9). However, at 2 × 10^−6 ^M Fe, microalgae that were grown axenically and in the phototrophic community lacked a significant difference in their growth parameters (*p* = 0.157 for r, *p* = 0.551 for k, and *p* = 0.444 for auc).

Although algal cell density increased at high Fe concentration (Fig. 5A), the reductive iron uptake mechanism was inversely proportional to the increase in iron concentration (Fig. 5B). Ferrireductase activity assesses Fe^2+^ on the cell surface. A higher ferrireductase activity indicates the reduction of Fe^3+^ in Fe^3+^-chelates to Fe^2+^ by algae. In both axenic and consortium setups, the membrane-bound ferrireductase activity of *C. sorokiniana* was reduced with increasing iron concentration (Fig. 5B) [65]. However, siderophore-producer *R. pickettii* PW2 significantly increased the ferrireductase activity in microalgae but only at lower Fe concentrations (1 × 10^−7 ^M Fe: *p* = 0.001, and 1 × 10^−6 ^M Fe: *p* = 0.003) (Table S10). At the same time, there was no observable ferrireductase activity in the axenic bacterial cultures. Therefore, azo dye reduction and iron reduction mechanisms in *C. sorokiniana* showed an inverse relationship with iron concentration.

Algae do not produce siderophores, however, they accumulate iron in the phycosphere by biosorption and chelation onto extracellular polymeric substances, including mono- and polysaccharides [66]. In contrast to the axenic culture, the EPS from *C. sorokiniana* co-cultured with bacteria accumulated significantly more iron (*t*-test; *p* < 0.05) (Fig. 5C). However, the difference in accumulation was significant only at low Fe concentrations: 1 × 10^−7 ^M and 1 × 10^−6 ^M, indicating iron accumulation was a stress response (Fig. S9) [67]. Iron accumulation at the surface acts as a signal for ferric-assimilating proteins (FEA1 and FEA2) to assimilate the chelated iron for intracellular uptake via a ferrireductase-dependent reductive pathway (Fig. S9). Such an iron uptake pathway has been reported in marine microalga *Chromera velia* [68] and freshwater alga *Chlorella sorokiniana* UTEX 1602 (Tables S13 and S14). In marine and freshwater algae such as *Scrippsiella trochoidea* [37], *Phaeodactylum tricornutum* [69, 70], and *Chlamydomonas reinhardtii* [71], plasma membrane-bound ferrireductase reduces Fe^3+^ chelated with siderophore to bioavailable Fe^2+^ (Fig. S9, Table S14). Different reductive pathways of Fe^2+^ transportation inside cells have been reported, such as reductive multicopper ferroxidase (FOX1) in *C. reinhardtii* [67] or via engulfment through IRT1/2 and NRAMP4 proteins in *C. reinhardtii* and *Ostreococcus tauri* [72]. In contrast, an endocytosis-mediated non-reductive pathway in which the whole Fe^3+^-siderophore is engulfed has also been reported in a marine alga *Phaeodactylum tricornutum* [37, 73]. Therefore, in this work, despite a considerable increase in microalgal growth in the consortium at a high Fe concentration, the ferrireductase activity lacked any significant increase (Fig. 5B), indicating a potential shift in an iron-uptake mechanism. At higher Fe concentrations, bacteria use ferric iron via direct diffusion across the cell membrane than high-affinity iron uptake by siderophore production and iron chelation [58, 74]. Like bacteria at high iron availability, microalgae uptake iron directly by a non-reductive direct iron-uptake pathway triggered due to the difference in intracellular and extracellular iron concentration (Fig. S9) [67, 75]. Thus, the selection of reductive or nonreductive pathways depends on both the external concentration of iron and its bioavailability [70].

**Fig. 6:**
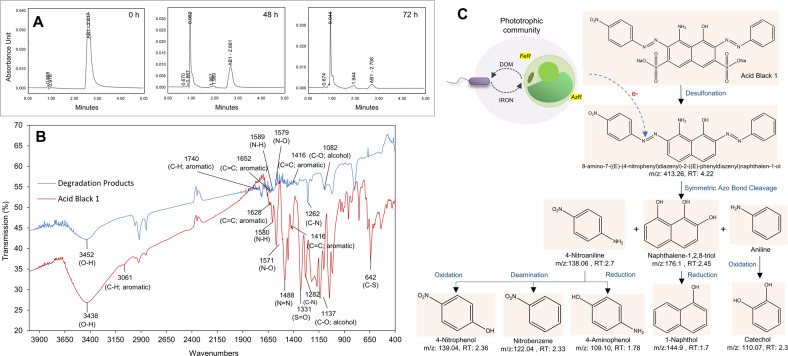
Azoreductase-mediated degradation of AB1 dye. **A** UPLC analysis suggests the disappearance of the peak (RT 2.617) of Acid Black 1 (AB1) dye after treatment with the phototrophic community. **B** In FT-IR spectra of AB1 dye and degradation products, the disappearance of vibrational bands at 1488 cm^−1^ and 1282 cm^−1^ suggests azoreductase-mediated cleavage of azo bond (-N = N-) [13]. **C** LCMS analysis of degradation products confirms the azoreductase-mediated symmetrical cleavage of azo bond into multiple by-products [13].

To further examine the effect of ferrireductase in bioremediation, microalgal cells were pretreated with 50 µM Diphenyleneiodonium (DPI), a known inhibitor of ferrireductase. DPI inhibits ferrireductase activity by preventing the transfer of an electron from ferrireductase (flavohemoproteins; Fre1) to the Fe-chelate, which obstructs the reduction of Fe^3+^ to Fe^2+^. Pretreatment of algal cells with 50 µM DPI significantly reduced the ferrireductase activity (*p* = 0.00) (Fig. 5D, Table S11) [75–77]. To further assess the linkage between ferrireductase and azo dye degradation, *C. sorokiniana* was treated with DPI and azoreductase activity was monitored. DPI (50 µM) treatment significantly inhibited azoreductase activities in algal cells both in the presence (Fe + DPI + ) (*p* = 0.008) and absence (Fe-DPI + ) (*p* = 0.035) of supplemented iron (Table S12). However, *C. sorokiniana* not pretreated with DPI (Fe-DPI-) retained significant azoreductase activity, which even increased when media was supplemented with iron (Fe + DPI-) (*p* = 0.012) (Fig. 5D). UPLC, FTIR, and LC-MS analyses further confirmed azoreductase-mediated degradation of AB1 dye by the microalgal-bacterial phototrophic community (Fig. 6A). In chromatograms, the peak at retention time (RT) of 2.617 corresponded to the reduction of AB1 dye. Similarly, in the FTIR analysis, the disappearance of vibrational bands at 1488 cm^−1^ and 1282 cm^−1^ suggested azoreductase-mediated cleavage of azo bond (-N = N-) in AB1 dye (Fig. 6B) [13]. The LC-MS confirmed the azoreductase-mediated AB1 dye degradation into 4-Nitroaniline (RT 2.7 min, m/z 138.06) and naphthalene-1,2,8-triol (RT 2.45 min, m/z 176.1) via symmetrical reductive cleavage of azo bonds (Fig. 6C, and S7, S8). Also, the presence of catechol (RT 2.3 min, m/z 110.07), a central intermediate of aerobic biodegradation of benzene derivatives, suggested mineralization of AB1 by-products via meta- or ortho-cleavage degradation pathways [78].

Azoreductases belong to oxidoreductases encompassing a diverse group of NADH or NAD(P)H cofactor-dependent flavoenzymes [18], which are involved in azo dye degradation as reported in numerous bacteria, fungi, and yeast [18, 79]. Recently, the dye decolorization potential of algae has been reported [80], but the involvement of azoreductase is not yet clear. We report a NAD(P)H mediated azoreductase activity in *C. sorokiniana* that is involved in azo dye degradation in completely photoautotrophic culture conditions. In our study, the ferrireductase-mediated reductive iron uptake mechanism in *C. sorokiniana* also influenced the azoreductase activity (Fig. 5D). A link between azoreductase and ferrireductase has previously been demonstrated in *Saccharomyces cerevisiae* [79]. In eukaryotes, three types of ferric reductases have been reported, i.e., NADPH oxidases (NOX; cytochrome b_558_), cytochrome b_5_ reductases, and cytochrome b_561_ [67]. The Fre1p (Ferric reductase 1) NOX enzyme encoded by the metalloregulator ferrireductase FRE1 gene in *S. cerevisiae* is responsible for 80–98% extracellular ferric reduction. The authors reported that the FRE1-dependent ferric reduction, inversely regulated by extracellular iron concentration, also participated in extracellular azo dye reduction. A gene knockout study in *S. cerevisiae* suggested the dependency of azoreductase on ferrireductase since deletion of FRE1 gene resulted in decreased dye degradation, which was restored when *S. cerevisiae* mutant cells were transformed with plasmid pSP3. Such transmembrane ferric-chelate reductases (FRE1) have also been reported from green microalga *Chlamydomonas reinhardtii* [71], which share high sequence similarity with ferric-chelate reductase protein from another microalga *C. sorokiniana* UTEX 1602 (Fig. S10, Table S13 and S14).

Although iron capture and transport mechanisms have been investigated in several single-cell eukaryotes [67, 70], the role of iron bioavailability in biotechnological applications has been largely unexplored. We demonstrate that when *C. sorokiniana* cells were treated with ferrireductase inhibitor DPI, the azoreductase-mediated dye degradation was inhibited (Fig. 5D). Since both enzymes function on an externally directed plasma membrane redox system, we propose that the ferrireductase-mediated reductive iron uptake mechanism in *C. sorokiniana* is also vital for azo dye degradation (Table S13). Like azoreductase, the ferric reductase pathway uses cytosolic NAD(P)H as an electron donor to transfer an electron to Fe^3+^ in ligand-bound complexes to release bioavailable Fe^2+^ [67]. Thus, highly diverse ferrireductase in algae can also reduce azo dyes externally. Additionally, iron acts as a redox catalyst in algae and mediates the electron transport reactions. Iron limitation can suppress the photosynthetic electron transfer in algae, reducing NAD(P)H formation [81]. Since the azoreductase mechanism is also a redox reaction mediated by the transport of electrons via NAD(P)H, thus, the reduced dye degradation activity, as observed in our study, could be due to the suppressed electron transport mechanism because of low iron. In contrast, the siderophore-mediated increase in iron bioavailability may have led to an increase in an electron transport mechanism and, consequently, azo reduction. However, further investigations using radiolabeling, omics, and expression-based studies of the oxidoreductases enzymatic machinery in *Chlorella* reveal the exact role of extracellular iron concentration in driving iron uptake via ferrireductase and dye degradation. Unlike bacteria, azoreductase in algae is relatively unexplored and requires further investigation.

A poor understanding of the extracellular environment, especially the role of micronutrients, has posed a major limitation in the industrial translation of single-species bioremediation designs. A synthetic community of algae and bacteria can be employed to understand several environmental interactions and overcome the challenges of conventional bioremediation designs. This work demonstrated that extracellular iron concentration influenced the ferrireductase-mediated iron uptake in *C. sorokiniana*, which also affected the dye reduction pathway. Bacterial siderophores have a major influence on iron cycling and shaping phototrophic communities in aquatic ecosystems [45]. In the high-nutrient, low-carbon (HNLC) regions of open oceans, bacterial siderophores have been known to alleviate iron limitation, which benefits algae [69, 82]. Similarly, regulation of iron bioavailability by bacterial siderophores in industrial setups can be used as a strategy to enhance algal growth and enzymatic activities. Synthetic algal-bacterial community designs can be replicated in various wastewater treatment processes or industrial bioprocessing setups characterized by iron and carbon limitations. Algal-bacterial consortium has been used to produce high-value bioproducts like lipids, proteins, vitamins, etc. [83], suggesting the benefit of synthetic community designs over single-species designs. Consortium design has been suggested to improve the growth of algal biomass and bring down operational costs [84]. Algal-bacterial designs will also help overcome the requirement of external chelators, such as EDTA.

Besides iron, external oxygen concentration also influences dye degradation, especially in conventional bacterial processes used in the textile industry. Azoreductase-mediated dye degradation in bacteria is a two-step redox process [13]. The primary reduction step requires oxygen-limiting conditions to reduce azo dyes via the azoreductase pathway. Following this, the secondary oxidation step requires well-oxygenated conditions to degrade by-products via oxygenases enzymes. Unlike bacteria, *Chlorella* can modify extracellular oxygen concentration in contrasting dark and light cycles to accelerate dye degradation [29]. It has been reported that dark cycles due to oxygen deprivation favor azoreductase, and light cycles due to oxygenated conditions favor degradation of dye by-products. Oxygenation by *Chlorella* also favors bacterial growth and ensures BOD/ COD reduction [85]. In closed photobioreactor-based designs, several studies on algal-bacterial respiration, COD removal, and nitrification have already highlighted the potential of such inter-kingdom symbionts in replacing monoculture processes in the treatment of industrial wastewater [85]. However, in an open system design, the stability of such symbionts can be challenged by co-existing microbes competing for resources. The stability of synthetic community designs can be ensured by investigating the ecological dynamics of phototrophic communities and identifying key factors responsible for associations, such as a preferred monosaccharide, chemotactic and signaling molecules, quorum sensing, vitamins, and C/N ratio [86, 87]. Regulation and monitoring of such key factors in a bioremediation setup will ensure an exclusive metabolic niche of the synthetic community and provide tolerance to environmental perturbation. Therefore, in addition to iron and carbon-assisted mutualism in *C. sorokiniana* and *R. pickettii*, further analysis of the underlying factors influencing microbial interactions would determine the stability of the synthetic community. The use of the systems biology approach would also enhance our understanding of abiotic and biotic interactions for designing synthetic communities for specific biotechnological applications [88].

**Fig. 7:**
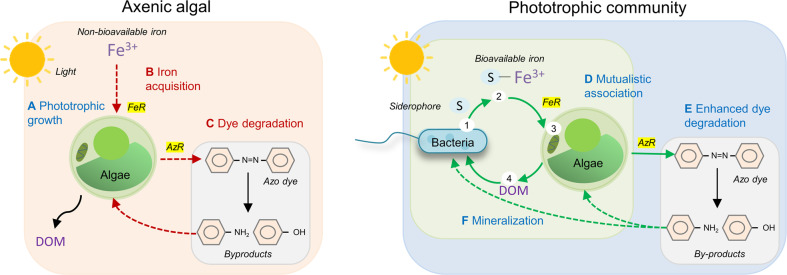
Proposed advantages of working with an algal-bacterial phototrophic community in comparison to axenic algal treatment setups for remediation of dyes. In a phototrophic textile wastewater treatment setup (**A**), algae have to perform multiple tasks like iron acquisition (**B**), and extracellular degradation of toxic dyes (**C**), which leads to metabolic burden (*dashed red lines*) and affects algal growth. Algae has a poor iron uptake mechanism, however, a consortium between algae and siderophore producing bacteria increases the bioavailability of iron for algae and reduces the metabolic burden (*green solid lines*). The mutualistic association between algae and bacteria allows the exchange of nutrients like iron, vitamins, Dissolved Organic Matter (DOM) (**D**) [37]. The bacterial-secreted siderophore chelates non-bioavailable Fe^3+^ and increased the iron bioavailability for ferrireductase (FeR) mediated uptake. The siderophore-mediated increase in iron bioavailability also increases ferrireductase activity, and thereby, algal growth. Algae, on the other hand, provides DOM for the sustenance of bacteria (step 4) [1]. From the study on *Chlorella sorokiniana* and *Ralstonia pickettii* PW2, the increase in bioavailability of iron also influenced the extracellular azoreductase (AzR) mechanism (**E**). The experimental evidence from this study suggests that the bacteria enhanced the algal ferrireductase and azoreductase activity, thus highlighting the potential link between these two enzymes [79]. Therefore, a consortium can decolorize the dye and further mineralize the degradation products (**F**).

In conclusion, the phototrophic community of *Chlorella sorokiniana* and *Ralstonia pickettii* represents a mutualistic association based on the exchange of specific limiting nutrients (Fig. 7). Our results form a basis for investigating a barter system between algae and bacteria, relying on an iron exchange from *R. pickettii* to *C. sorokiniana* and dissolved organic matter from *C. sorokiniana* to *R. pickettii*. Under iron stress, bacterial siderophore ensures iron availability for the algal partners, promoting the algal growth rate and potential to degrade dye. Therefore, the bacterial-algal association has the potential to treat industrial wastewater having carbon and iron limitation. We report that the transmembrane ferrireductase activity in *C. sorokiniana* plays a crucial role in the reductive iron-uptake mechanism that triggers azoreductase activity. Bioavailable iron regulates the activity of both oxidoreductase enzymes, enhancing dye degradation by *Chlorella*. Therefore, a microalgal-bacterial consortium working under photoautotrophic conditions could provide a self-sustainable alternative to current monoculture remediation processes. It would be worth investigating whether increased iron bioavailability also improves the ability of the microalgal-bacterial consortium to remediate other organic pollutants, which require extracellular reductive cleavage.

## Materials and methods

### Culture media preparation, sample collection, and bacterial identification

Three freshwater algal strains, *Chlorella sorokiniana* (CCAP 211/8 K), *Scenedesmus* sp., and *Oscillatoria animalis* (Sciento Ltd), were cultivated in Bold’s Basal Medium (3N-BBM + V; henceforth referred to as BBM) and maintained with EDTA-chelated iron at 28 °C under continuous white light at 80 μmol photons m^−2^ s^−1^ from fluorescent lamps (Fig. 1B). The NaNO_3_ in BBM media served as the sole nitrogen source for algal growth. Two strains of the marine microalga *Phaeodactylum tricornutum* 1052/6 and 1055/1, were grown on an *f/2* medium [89]. Unless specified, 1 × 10^−6 ^M FeCl_3_.6H_2_O was used as an iron source. However, iron-abundant BBM was referred to as BBM + Fe (chelated with 10-folds Na-EDTA) and iron-deficient as BBM-Fe (not EDTA-chelated), with the same Fe concentrations. Here, ‘+Fe’ represents more bioavailability of Fe due to chelation due to EDTA, and ‘-Fe’ represents low bioavailability of Fe.

Textile wastewater was collected from Panipat, India (29.363121 N, 76.992971 E). The non-selective 10% tryptone soya agar (TSA) was used to isolate bacteria from wastewater [90] (Fig. 1A). The morphologically distinct colonies were isolated, and genomic DNA was purified using Wizard Genomic DNA Purification Kit (Promega, USA). 16 S rRNA gene was amplified using PCR with universal primers 27 F and 1492 R for 30 cycles and purified using QIAprep Miniprep Kit (Qiagen, Netherlands) [13, 91]. The 16 S rRNA sequencing reactions were performed by the Sanger sequencing technique using 785 F and 907 R primers. The resulting sequences were checked for low-quality reads and overlaps using Finch TV software (version 1.4.0). The homologous sequences were searched in the NCBI GenBank database using blastn feature, and the bacterial isolates were identified by comparing their percent identity scores. The sequences were submitted with accession numbers MW857264-70. For further experiments, the isolates were revived in 10% tryptone soya broth (TSB) (Fig. 1A).

### Bacterial siderophore production and algal dye degradation assessment

Bacterial siderophore production was estimated using standard Chrome Azurol S (CAS) assays in modified Minimal Medium 9 [74, 92] (Fig. 1C). Siderophore production was ascertained as a yellow/orange zone around bacterial colonies on the CAS agar plate. Siderophore was quantified using CAS liquid assay using Desferrioxamine mesylate as standard and further categorized using Csaky’s assay (for hydroxamate) with Desferrioxamine mesylate and Arnow’s assays (for catecholate) with 2’3-Dihydroxybenzoic acid as standards [74]. All the experiments were performed in triplicates.

Microalgal dye degradation potential was assessed using Acid Black 1 dye (AB1, Sigma Aldrich; CAS: 1064-48-8) (Fig. 1D). Ten-day old freshwater and marine algae cultures were preincubated in EDTA-chelated growth media for 48 h before adding filter-sterilized (0.2 µm) 10 µM AB1 dye. Microalgal cultures were incubated at 28 °C under continuous light for 72 h, and cell biomass was removed by centrifuging the culture at 5000 × *g* for 5 min. The supernatant was used to estimate the dye decolorization by microalgal cultures by calculating the per cent dye removal [13]. All the experiments were performed in triplicates.

### Algal-bacterial co-culturability assessment under iron limiting conditions

To analyze the nature of algal-bacterial interactions in an iron-limiting environment, the screened dye-decolorizer microalgae were co-cultured with siderophore-producing bacteria, and their growth characteristics were determined. An overnight bacterial culture raised in 10% TS broth was washed thrice with sterile BBM-Fe. Bacterial suspension in fresh BBM-Fe (OD_600nm_ = 0.3) was inoculated in microalgal exudates obtained by filter sterilization (0.2 µm) of 7-day old algal culture raised in BBM + Fe. The bacterial growth (OD_600nm_) was taken as a measure of the potential of bacteria to use algal-derived dissolved organic matter (DOM) as a carbon source (Fig. 1E).

Bacterial isolates showing growth in microalgal exudates were selected to ascertain their co-culturability [37] (Fig. 1F). Overnight cultures of bacteria raised in BBM + Fe supplemented with 0.1% glucose were washed with sterile BBM-Fe, and 1 ml of bacterial suspensions (OD_600_ = 0.5) were inoculated in 20 ml of 1-day old cultures of microalgae raised in BBM-Fe media [44, 58]. Growth characteristics of both microalgal and bacterial co-inoculants were observed to determine the mutualistic/antagonistic/neutral interactions. Bacterial and algal growth were determined periodically over 12 days on TSA plates (CFUs) and under the microscope using a hemocytometer, respectively [37]. The algal growth curve was fitted in the logistic equation to determine the growth parameters (growth rate ‘r’, carrying capacity ‘k’, doubling time ‘Dt’, and area under curve ‘auc’) using the growth prediction modeling package ‘*growthcurver*’ in R [42]. All the experiments were performed in triplicates.

The difference in algal growth in axenic and co-culture setup was analyzed statistically using One-Way ANOVA and Tukey’s post-hoc test in SPSS 16.0. The Principal Component Analysis (PCA) was applied to growth parameters using ‘*FactoMineR*’ package in R. Bacterial and microalgal isolates showing mutualistic associations were designated as a ‘consortium/ phototrophic community’ for further experiments on azo dye degradation (Fig. 1G).

### Characterization of carbohydrates in algal exopolysaccharides (EPS)

The EPS from *C. sorokiniana* and *Scenedesmus* sp. were fractionated using Dowex Marathon C cation exchange resin (Sigma Aldrich, USA) [93]. The algal cells from a 7-day old culture in BBM-Fe were washed and centrifuged at 2000 × *g* for 3 min at RT. The cell pellets were resuspended in PBS buffer containing Dowex Marathon (25 g g^−1^) and gently mixed in a rotatory mixer (50 RPM) at 4 °C for 1 h. Subsequently, the algal cells were centrifuged at 4000 × *g* for 4 min, and the supernatant was subjected to overnight precipitation of exopolysaccharides in 70% ethanol (3:1 to supernatant) at 4 °C [94]. After that, the supernatant was centrifuged at 10,000 × *g* for 10 min, and the pellet was collected for analyses by high-performance anion-exchange chromatography (HPAEC). Pellets were acid hydrolyzed with 72% H_2_SO_4_ at 121 °C for 30 min. Monosaccharides present in hydrolyzed EPS were identified using Ion Chromatography System (Thermo Scientific Dionex ICS 5000 + , UK) with AminoPac PA10 column (250 mm × 4 mm) and a pulsed amperometric detector. KOH (1 mM) was used for isocratic elution and separation at 0.25 mL min^−1^ for 25 min. As standards, arabinose, fructose, galactose, glucose, mannose, and rhamnose were used. To understand the carbon preference, screened bacterial strains were cultured in individual sugars (0.1% concentration) supplemented in BBM media. All the experiments were performed in triplicates, and the concentrations of sugars in EPS were reported in g gcell^−1^.

### Dye degradation potential of the phototrophic community under varying environmental conditions

To evaluate the performance of the phototrophic community in degrading dye, three experimental setups in BBM + Fe and BBM-Fe were conducted: (i) Setup 1: axenic alga, (ii) Setup 2: algal-bacterial consortium, and (iii) Setup 3: axenic bacteria (with 0.01% glucose) (Table S3). The microalgal cells, previously grown in BBM + Fe under static conditions at 28 °C in continuous light, were washed and used in all setups. FeCl_3_ (1 × 10^−6 ^M) was supplied to maintain the iron-deficient environment and 20 µM AB1 dye for decolorization assessment. The dye decolorization was assessed by obtaining AB1 concentration over a time interval of 144 h [13]. The rate kinetics was determined by fitting the concentration data in multiple degradation models like Single First Order (SFO) and First Order Multi-Compartment (FOMC) using ‘*mkin*’ package [95, 96]. The chi-square (χ^2^) statistic was used to determine the goodness of fit of the kinetic models. The variation in the rate of AB1 degradation was measured using Levene’s, One-way ANOVA, and Tukey’s posthoc tests in SPSS 16.0. All the experiments were performed in triplicates.

Further, the influence of environmental factors pertinent to textile wastewater in algal dye degradation was studied using a multi-factor design based on Taguchi’s L_16_ (4^3^) orthogonal array. Thirty-two experiments were conducted in Setups 1 and 2 in BBM-Fe media (not EDTA-chelated) with varying concentrations of Fe, AB1 dye, and pH computed using Minitab ver.19 [97]. Details of variables/levels were as follows: Fe: 1 × 10^−7^, 1 × 10^−6^, 2 × 10^−6^, and 5 × 10^−6 ^M; pH: 6.0, 7.0, 8.0, and 9.0, and (iii) dye: 4, 8, 12, and 16 µM (Table S5). Microalgal cells were previously starved for iron in BBM-Fe media for 24 h at various pH levels as per the L_16_ design (Table S5). For experiments in setup 2, a microbial consortium was developed by mixing an overnight culture of siderophore-producer bacterial isolate (OD = 0.3) with microalgal cells (1% v/v). The culture was spiked with different concentrations of sterile iron (FeCl_3_.6H_2_O) and AB1 dye as per the L_16_ design and maintained under static conditions at 28 °C with continuous light (Table S5). Uninoculated BBM-Fe was kept as a control. Microbial cells were carefully separated by centrifugation at 4000 × *g* for 4 min, and culture media was sampled periodically over 48 h; dye concentration was determined in different experimental setups [13]. The rate of AB1 degradation was calculated by fitting the concentration data in the first-order kinetic model using ‘*mkin*’ package in R [95, 98]. The variations in dye degradation in different setups were analyzed by a multiple linear regression model using Minitab ver.19. The effect of the interaction between the factors (Fe*pH, Fe*Dye, and pH*Dye) on the rate of dye degradation was assessed by performing Partial Least Squares Path (PLSP) modeling of the L_16_ design in R [99]. All the experiments were performed in triplicates.

### Assessment of algal ferrireductase, azoreductase activity, and iron concentration

The ferrireductase activity was determined in microalgae raised axenically and in consortium under different iron concentrations (FeCl_3_.6H_2_O: 1 × 10^−7^, 1 × 10^−6^, and 2 × 10^−6 ^M). The growth characteristics of microalgal cells were observed using the ‘*growthcurver*’ package. After 10 days of incubation, microalgal cells were harvested, washed, and resuspended in PBS. An equal number of microalgal cells were maintained in all the assays [37]. The reaction mixture contained PBS with 130 µM HEPES (pH 8.1), 10 µM Fe (with 10-folds EDTA), and 100 µM bathophenanthrolinedisulfonic acid (BPDS), i.e., Fe^2+^ chelator. The ferrireductase activity was estimated as the formation of Fe^2+^-BPDS complex at OD_535nm_ using the extinction coefficient 22140 M^−1^cm^−1^.

Simultaneously, the cellular concentration of iron in microalgae was estimated using ICP-MS. The culture was centrifuged at 4000 × *g* for 4 min, and the cells were removed, washed, and resuspended in sterile deferrated BBM. Microalgal cells were acid digested in 70% HNO_3_ and appropriately diluted for estimating iron content by ICP-MS (Bruker M90 ICP-MS). To estimate iron on the cell surface, microalgal EPS was extracted as described previously. The EPS was also digested in 70% HNO_3_ and diluted for estimating the Fe by ICP-MS. FeCl_3_ was used as a standard.

The link between azoreductase with ferrireductase enzymes in microalgae was determined using Diphenyleiodonium (DPI), an inhibitor of ferrireductase [76, 77]. The optimum concentration of DPI was determined by a ferrireductase assay using microalgal cells pretreated with different concentrations of DPI (50, 100, and 150 µM) for 1 h. Excessive DPI was removed by a thorough washing of the cells, and an azoreductase assay was carried out in a reaction buffer containing 50 mM phosphate buffer (pH 7.2) and 10 µM AB1 dye [62]. The reaction was initiated by adding 0.5 mM NAD(P)H. The enzyme activity was determined by a decrease in OD_618nm_ over 240 min. Azoreductase activity was monitored at varying conditions of Fe (EDTA-chelated) and DPI concentration, i.e., Fe-DPI-, Fe+DPI-, Fe-DPI + , and Fe+DPI + . Linear regression model and One-Way ANOVA were used to analyze variations in the enzyme activities in different experimental setups using SPSS 16.0 and R. All the experiments were performed in triplicates.

### Assessment of AB1 degradation pathway

Microbial consortia cultivated in BBM-Fe were challenged with AB1 dye under iron stress of 1 × 10^−6 ^M FeCl_3_. Aliquots of culture were sampled aseptically by removing the microbial cells by centrifuging at 10000 × *g* for 15 min. The supernatant was filtered through a 0.2 µm filter, and the filtrate was analyzed by UPLC (Waters Acquity UPLC system, United States) using BEH C18 100 mm×2.1 mm column fitted with a photodiode array detector. Acetonitrile with 0.2% formic acid in water (85:15 v/v) was used as a mobile phase, and the peaks were recorded at OD_595nm_. AB1 dye was used as a standard.

The AB1 degradation products in the filtrate were extracted with an equal volume of ethyl acetate and subjected to FTIR analysis, as mentioned previously [13]. The biodegraded products were identified using LC-MS analyses (Dionex Ultimate 3000, Thermo Scientific; Q Exactive Orbitrap Mass Spectrometer, Thermo Scientific) with acetonitrile: water (70:30) as a solvent system in isocratic elution mode (refer to supplementary Method S1 for details). The sample dissolved in HPLC grade methanol was injected in Hypersil Gold (5 µm, 100 cm×2.1 µm) column for a run time of 20 min at a positive polarity (+1) mode. The mass spectra were recorded within 100–1200 m/z with a maximum ion transfer time of 100 ms. Degraded products were identified by analyzing mass fragment peaks and referring to NIST and MassBank libraries, and existing literature.

## Supplementary information

Supplementary Material

## Data Availability

The 16 S rRNA sequences have been submitted to the NCBI GenBank under the accession numbers MW857264-70. All data has been included in the MS and supplementary files.
