# Supplementary material for: Iron-dependent mutualism between Chlorella sorokiniana and Ralstonia pickettii forms the basis for a sustainable bioremediation system
Source: ISME Commun. 2022 Sep 15;2:83. doi: 10.1038/s43705-022-00161-0 (PMC9476460; doi:10.1038/s43705-022-00161-0)
Supplement: Supplementary file 1 — Supplementary Material [file 43705_2022_161_MOESM1_ESM.docx]

**Supplementary Material**

**Iron-dependent mutualism between *Chlorella sorokiniana* and *Ralstonia pickettii* forms the basis for a sustainable bioremediation system**

Deepak Rawat^1,2,3^, Udita Sharma^1^, Pankaj Poria^1^, Arran Finlan^2^, Brenda Parker^2,*^, Radhey Shyam Sharma^1,4,*^, Vandana Mishra^1,*^

**Affiliations**

^1^ Bioresources & Environmental Biotechnology Laboratory, Department of Environmental Studies, University of Delhi, Delhi-110007, INDIA

^2^ Department of Biochemical Engineering, Bernard Katz Building, University College London, Gower Street, London WC1E 6BT, UK

^3^ Department of Environmental Studies, Janki Devi Memorial College, University of Delhi, Delhi-110060, INDIA

^4^ Delhi School of Climate Change & Sustainability, Institute of Eminence, University of Delhi, Delhi-110007, INDIA

**Corresponding Authors (*)**

1. Prof. Vandana Mishra, Bioresources & Environmental Biotechnology Laboratory, Department of Environmental Studies, University of Delhi, Delhi-110007, INDIA, +91 98712 60287, [mistletoe_h@hotmail.com](mailto:mistletoe_h@hotmail.com) (ORCID ID: 0000-0002-8173-9207)
2. Dr Brenda Parker, Department of Biochemical Engineering, Bernard Katz Building, University College London, Gower Street, London WC1E 6BT, UK, 02076799789, [brenda.parker@ucl.ac.uk](mailto:brenda.parker@ucl.ac.uk) (ORCID ID: 0000-0002-4869-9637)
3. Prof. Radhey Shyam Sharma, Bioresources & Environmental Biotechnology Laboratory, Department of Environmental Studies, University of Delhi, Delhi-110007, INDIA, +91 98102 27222, [rads26@hotmail.com](mailto:rads26@hotmail.com) (ORCID ID: 0000-0003-1397-9230)

1. Supplementary Data

**Data S1: Assessment of siderophore production in bacteria and dye degradation in algae**

Out of seven bacterial isolates purified from the untreated textile wastewater (Table 1), five showed relatively high siderophore production (Supplementary Fig. S1). *Ralstonia pickettii* PW2, *Serratia plymuthica* PW1, and *S. liquefaciens* PW71 grew within 24 h on deferrated CAS agar plates and showed siderophore activity [1]. *Stenotrophomonas maltophilia* PW5 and *S. maltophilia* PW6 showed high growth and siderophore production but only after 96 h of incubation. *Stenotrophomonas rhizophila* PW3 and *Stenotrophomonas rhizophila* PW72, however, failed to grow on CAS agar plates. *S. plymuthica* PW1, *S. liquefaciens* PW71, and *R. pickettii* PW2 produced siderophore in decreasing order of concentration, i.e., 15.26±1.3 > 13.28±0.9 > 10.85±0.7 µMmL^-1^ (Table 1). Arnow’s and Csaky’s assay confirmed a catecholate-type siderophore is produced by *Serratia plymuthica* PW1 (81.10±9.8 µMmL^-1^), *Ralstonia pickettii* PW2 (97.43±16.8 µMmL^-1^), and *Serratia liquefaciens* PW71 (103.1±8.3 µMmL^-1^). On the other hand, hydroxamate-type of siderophore is produced by *S. maltophilia* PW5 (37.86±0.4 µMmL^-1^) and *S. maltophilia* PW6 (17.73±0.2 µMmL^-1^) (Table 1). As *S. rhizophila* PW3 and *S. rhizophila* PW72 showed low culturability in iron-limiting conditions, they were omitted from further experiments.

*S. plymuthica* PW1 and *S. liquefaciens* PW71 from textile dye effluent produce catecholate siderophores (Table 1), common among stress-tolerant bacterial genera [2]. Different *Serratia* species thrive well in contaminated environments such as textile dye effluent and petroleum [3]. *R. pickettii* PW2, a β-proteobacterium, has also been reported to produce siderophores under iron stress [4].

Out of the five algal species screened, freshwater microalgae *Chlorella* *sorokiniana*, *Scenedesmus* sp., and freshwater cyanobacterium *Oscillatoria* *animalis* were observed to degrade AB1 dye. After 72 h, the AB1 degradation potential of microalgae was in decreasing order as *C. sorokiniana* (82.10±1.6%) > *Scenedesmus* sp. (31.04±3.1%) > *O. animalis* (30.36±1.8%). The marine algal species *Phaeodactylum tricornutum* 1052/6 and 1055/1 did not decolorize AB1 dye under the conditions tested. *Chlorella* and *Scenedesmus* sp. have also been shown to remediate a wide range of pollutants such as textile dyes [5, 6], heavy metals [7], pesticides [8], and aromatic hydrocarbons [6]. Therefore, we selected *Chlorella* and *Scenedesmus* for testing and developing a synthetic phototrophic community.

**Data S2: Iron and carbon dependent mutualism between *Chlorella sorokiniana* and *Ralstonia pickettii***

The exudates from *C. sorokiniana* and *Scenedesmus* sp. were used as a source of dissolved organic matter for cultivating bacteria and identifying suitable microalgal-bacterial combinations for the phototrophic community (Fig. 1E). All five bacterial isolates grew well on the exudate of *C. sorokiniana* as a sole source of carbon. On the contrary, on exudates of *Scenedesmus* sp.*, S. plymuthica* PW1 showed moderate growth in 20 h while the growth of *R. pickettii* PW2 and *S. liquefaciens* PW71 remained insignificant. *S. maltophilia* PW5 and *S. maltophilia* PW6 did not grow on the exudate of *Scenedesmus* sp., thus omitted from further experiments (Supplementary Fig. S2B).

Different combinations of phototrophic community with microalgal (*C. sorokiniana*/ *Scenedesmus* sp.) and siderophore-producer bacterial (*S. plymuthica* PW1/ *R. pickettii* PW2/ *S. liquefaciens* PW71) partners were assessed under iron limiting BBM media (BBM-Fe) without EDTA (Fig. 1F). In absence of EDTA, Fe precipitates rapidly as iron oxyhydroxides and becomes unavailable to the algae. Therefore, bacterial siderophore facilitates chelating iron making it bioavailable for both bacteria and algae. Microalgal cell count in consortium culture was compared with that of axenic microalgal culture to categorize its interaction with a bacterial partner as mutualistic, antagonistic, and neutral. In contrast with axenic microalgal culture (~4×10^6^ cells mL^-1^), *C. sorokiniana* in co-culture with *R. pickettii* PW2 showed a significant increase in cell count at 200 h (~6×10^6^ cells mL^-1^), suggesting a mutualistic association (auc; *p*=0.000) (Fig. 2A; Supplementary Table 2). However, *S. plymuthica* PW1 exerted an antagonistic effect on *C. sorokiniana* (auc; *p*=0.001), whereas, the interaction between *S. liquefaciens* PW71 and *C. sorokiniana* remained neutral (auc; *p*=0.430) (Fig. 2A, Supplementary Table S2). During early growth, the interaction between *Scenedesmus* sp. and *S. plymuthica* PW1 remained neutral; however, later, the interaction turned antagonistic (Fig. 2A). Whereas the interaction of *Scenedesmus* sp. with both *R. pickettii* PW2 and *S. liquefaciens* PW71 was neutral. *Scenedesmus* showed a higher growth (~12×10^6^ cells mL^-1^) than axenically grown *Chlorella* (~5×10^6^ cells mL^-1^), suggesting an effective iron-uptake mechanism under iron-limiting conditions (k; t-test, *p*=0.001) (Supplementary Table S1).

Growth parameters such as carrying capacity (k), growth rate (r), doubling time (Dt), and area under curve (auc) provided better insights into the population ecology of microalgae [9] (Supplementary Fig. S2, Table S1, and Table S2). As indicated by a steeper slope of log-phase in the growth curve (Fig. 3A), the growth rate of *C. sorokiniana* in consortium with *R. pickettii* PW2 (5.02±1.0×10^-2^ h^-1^) remained significantly higher than that of axenically grown microalgae (1.95±0.3×10^-2^ h^-1^) (*p*=0.000). However, the carrying capacity of *C. sorokiniana* remains unchanged with and without culturing with *R. pickettii* PW2 (co-culture: 4.97±0.1×10^6^ cells vs axenic culture: 4.82±0.4×10^6^ cells; *p*=1.000). In the consortium, *C. sorokiniana* showed a higher population turnover during the early log-phase and reached the stationary phase earlier (at 100 h) than that grown axenically (~270 h), though carrying capacity remained similar (Fig. 3A). The early stationary phase of *C. sorokiniana* in consortium culture could be due to faster utilization of iron, which was the sole growth-limiting factor. Such increased growth rate could also be due to faster N uptake, however, the NO_3_-N concentration in both axenic algal and consortium culture was ~75 mgL^-1^ after 14 days (initial concentration was 123 mgL^-1^), suggesting faster growth was due to higher bioavailability of iron. A significantly higher area under curve in the consortium (11.01±0.4×10^8^) of *R. pickettii* PW2 in comparison with its axenic culture (6.01±0.5×10^8^) (*p*=0.000) also indicates mutualism between *C. sorokiniana* and *R. pickettii* PW2 (Fig. 3A).

*S. plymuthica* PW1 exerted an antagonistic effect on *C. sorokiniana* as indicated by its significant increase in its doubling time (*p*=0.009) with lower growth rate, carrying capacity, and area under curve, compared with the axenic culture of microalga (Fig. 3A). However, the lack of significant difference in growth parameters of *C. sorokiniana* co-cultured with *S. liquefaciens* PW71 indicates a neutral relationship between the two (Supplementary Table S2). *S. plymuthica* PW1, *S. liquefaciens* PW71, and *R. pickettii* PW2 showed an antagonistic effect on *Scenedesmus* sp., though the degree of effect varied (Fig. 3A). Co-culture of *S. plymuthica* PW1 with *Scenedesmus* sp., for example, caused a significantly reduced carrying capacity (*p*=0.000) and area under curve (*p*=0.003) of *Scenedesmus* sp. (Supplementary Table S2). Among these bacteria, *S. plymuthica* PW1 was antagonistic to both the microalgae.

Biplot based on principal component analyses (PCA) of microalgal growth parameters in different experimental setups further explained the difference in the growth of *C. sorokiniana* and *Scenedesmus* sp. in axenic cultures and consortium with bacteria. In the PCA biplot, *Scenedesmus* sp. from three setups, i.e., axenically grown (SS), in consortium with *R. pickettii* PW2 (SSPW2), and *S. liquefaciens* PW71 (SSPW71) were grouped close to each other based on growth variables such as the area under curve (auc) and carrying capacity (k) metrices, whereas *Scenedesmus* in consortium with *S. plymuthica* PW1 (SSPW1) was separated from the group. In contrast, *C. sorokiniana* in all four setups i.e., axenically grown (CS), in consortium with *S. plymuthica* PW1 (CSPW1), *R. pickettii* PW2 (CSPW2), and *S. liquefaciens* PW71 (CSPW71) were separated from each other based on growth variables doubling time (Dt) and growth rate (r) (Fig. 3B). PC1 explains 54.7% of the grouping of different variables. The percent contribution of ‘auc’ was highest in PC1 (39.17%), followed by ‘Dt’ (25.36%), ‘k’ (24.39%), and ‘r’ (11.07%) (Supplementary Fig. S2D). The ‘auc’ metric represents the overall area under the growth curve and ‘k’ represents the maximum population growth, therefore, both the metrices represent the overall growth of the microalgae. In comparison to *Chlorella*, *Scenedesmus* had a higher growth under iron limiting conditions (Fig. 2A), therefore, *Scenedesmus* setups (SS, SSPW2, and SSPW71) were separated from *Chlorella* setups based on ‘auc’ and ‘k’. Also, there was no significant difference between SS, SSPW2, and SSPW71 based on growth parameters like ‘Dt’, ‘r’, and ‘k’ (Fig. 3A), therefore, all the setups were grouped close to each other. Whereas, *Scenedesmus* grown in presence of *S. plymuthica* PW1 (SSPW1) was separated from other *Scenedesmus* setups as there was a significant reduction in carrying capacity ‘k’ (Fig. 3A) due to the negative effect of the bacteria on algal growth. PC2 explains 37.7% of the grouping of different variables, which has ‘r’ as the dominant metric (42.25%), which explains most of the variance followed by ‘k’ (29.30%), ‘Dt’ (20.47%), and ‘auc’ (7.98%) (Supplementary Fig. S2D). *Chlorella* in presence of *R. pickettii* PW2 (CSPW2) showed a significant increase in growth rate ‘r’ and a significant reduction in doubling time (Dt) (Fig. 3A), therefore, CSPW2 was separated from axenic *Chlorella* setup (CS) along with growth parameter ‘r’.

The increasing growth rate of *C. sorokiniana* can be attributed to the increased iron bioavailability by siderophore-producing *R. pickettii* [10]. Such an iron-dependent mutualism between previously non-associated algae and bacteria has been shown between *Dunaliella bardawil* and *Halomonas* sp., where *Halomonas* sp. facilitates the iron in exchange for microalgal DOM [11]. Also, a cupriachelin siderophore is based between freshwater diatom *Navicula pelliculosa* and bacterium *Cupriavidus necator* [12]. In the marine environment, the vibrioferrin siderophore produced by *Marinobacter* sp. accelerates *Scrippsiella trochoidea* growth [13], and *Idiomarina loihiensis* siderophore promotes the growth of *Chlorella variabilis* [14]. On the contrary, *C. sorokiniana* grown with *S. plymuthica* PW1 (CSPW1) separated from axenic alga (CS) due to a higher ‘doubling time (Dt)’, which is also supported by the slower growth in consortium due to the negative effect of bacterial partner as observed from algal growth curve (Fig. 2A).

The HPAEC analyses of exopolysaccharides (EPS) of *C. sorokiniana* detected galactose (0.03±0.0 g gcell^-1^; 52% relative percentage) as a dominant monosaccharide besides glucose (0.01±0.0 g gcell^-1^, 20%), mannose (0.01±0.0 g gcell^-1^; 20%), arabinose (4%), and rhamnose (4%) (Fig. 2B and Supplementary Fig. S3A). *S. plymuthica* PW1 grew well in all five monosaccharides (0.1%), being galactose as the most preferred carbon source (Fig. 2C; Supplementary Fig. S3B). *R. pickettii* PW2 showed a preference for galactose followed by glucose and mannose, though the growth promoted was relatively lower than that observed in *S. plymuthica* PW1 (auc; *p*=0.000) (Supplementary Fig. S3B, Table S15). In *C. sorokiniana*, galactose has been reported previously as a dominant monosaccharide (67%). Galactose also serves as a signaling molecule in bacteria, and its presence in EPS has been hypothesized to extend the stationary phase in microalga *Botryococcus braunii* [15]. In contrast with *Chlorella*, *Scenedesmus* sp. showed a different sugar profile with glucose (37%) being the dominant monosaccharide followed by mannose (32%), rhamnose (12%), galactose (10%), and rhamnose (9%) (Fig. 2B). Bacteria grew well on *Chlorella* exudates rich in galactose than the exudates of *Scenedesmus* sp. rich in glucose and mannose (Supplementary Fig. S2B). The result suggest that the difference between in the sugar profile of EPS of the two algal species influence the growth of bacteria and also the microalgal-bacterial association [16].

Previously, a commensal association between a bacterium with a high percentage identity to *R. pickettii* and *C. sorokiniana* has been shown under nutrient-sufficient photoautotrophic conditions [17]. The co-occurrence of *R. pickettii* in the non-axenic culture of microalga *Botryococcus braunii* also suggests the environmental prevalence of the bacterium [18]. Our study suggests *Chlorella* EPS serves as a source of DOM for *R. pickettii* PW2, which influences the mutualistic association between them under the iron limitation. *R. pickettii* PW2 showed high growth when co-cultured with *C. sorokiniana*, suggesting its preference for algal EPS (Fig. 2A). Further assay on individual sugars suggested a high affinity of *R. pickettii* for galactose, which was produced more in *Chlorella* in comparison to *Scenedesmus* (Fig. 2B, 2C). Whereas, under experimental conditions, *S. plymuthica* PW1 showed ~10 times more growth than *R. pickettii* PW2 when co-cultured with *C. sorokiniana* (Fig. 2A). Such antagonistic effect by *S. plymuthica* PW1 on *C. sorokiniana* could be attributed to its aggressive growth (Fig. 2A). In comparison to *R. pickettii*, *S. plymuthica* PW1 grew in all five sugars, suggesting a generalist and competitive life strategy (Fig. 2C). The composition of algal exopolysaccharides determines the association with heterotrophic bacteria and community structure [19]. Bacteria also show high preference for algal-derived saccharides for carbon, which may play a vital role in initiating and maintaining such symbiotic associations [19, 20]. Thus, we hypothesize that the success of such algal-bacterial mutualistic associations could be governed by the composition of algal EPS and the affinity of bacteria for the monosaccharide as a preferred carbon source.

**Data S3: MS/MS peaks obtained from LCMS analysis**

| **Compound** | **RT** | **Description** | **Identified Peaks** |
| --- | --- | --- | --- |
| 1- Naphthol | 1.67 | Chemical Formula: C10H8O  Exact Mass: 144.06  Molecular Weight: 144.17  m/z: 144.06 (100.0%), 145.06 (10.9%) | 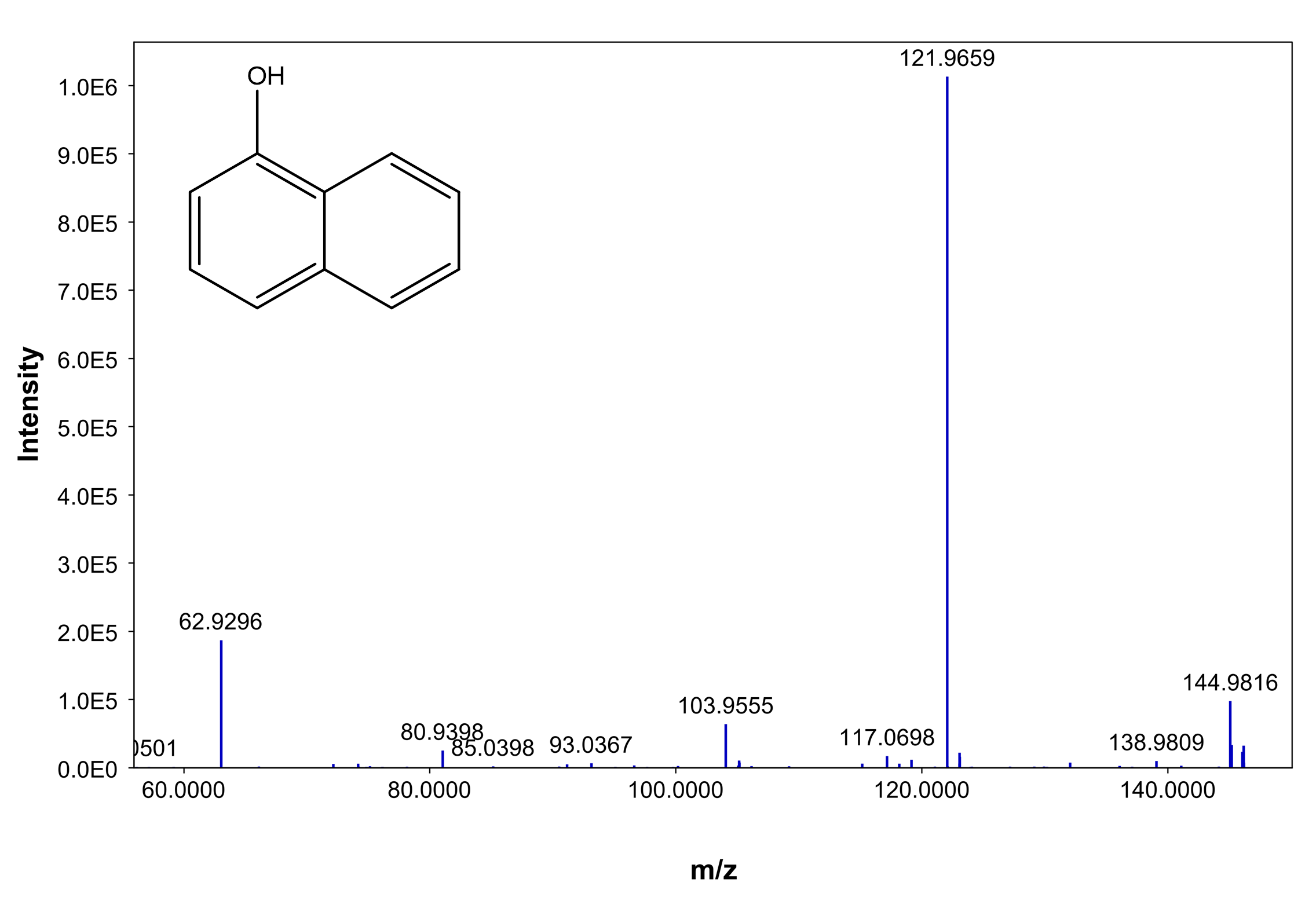 |
| 4-Aminophenol | 1.78 | Chemical Formula: C6H7NO  Exact Mass: 109.05  Molecular Weight: 109.13  m/z: 109.05 (100.0%), 110.06 (6.6%) | 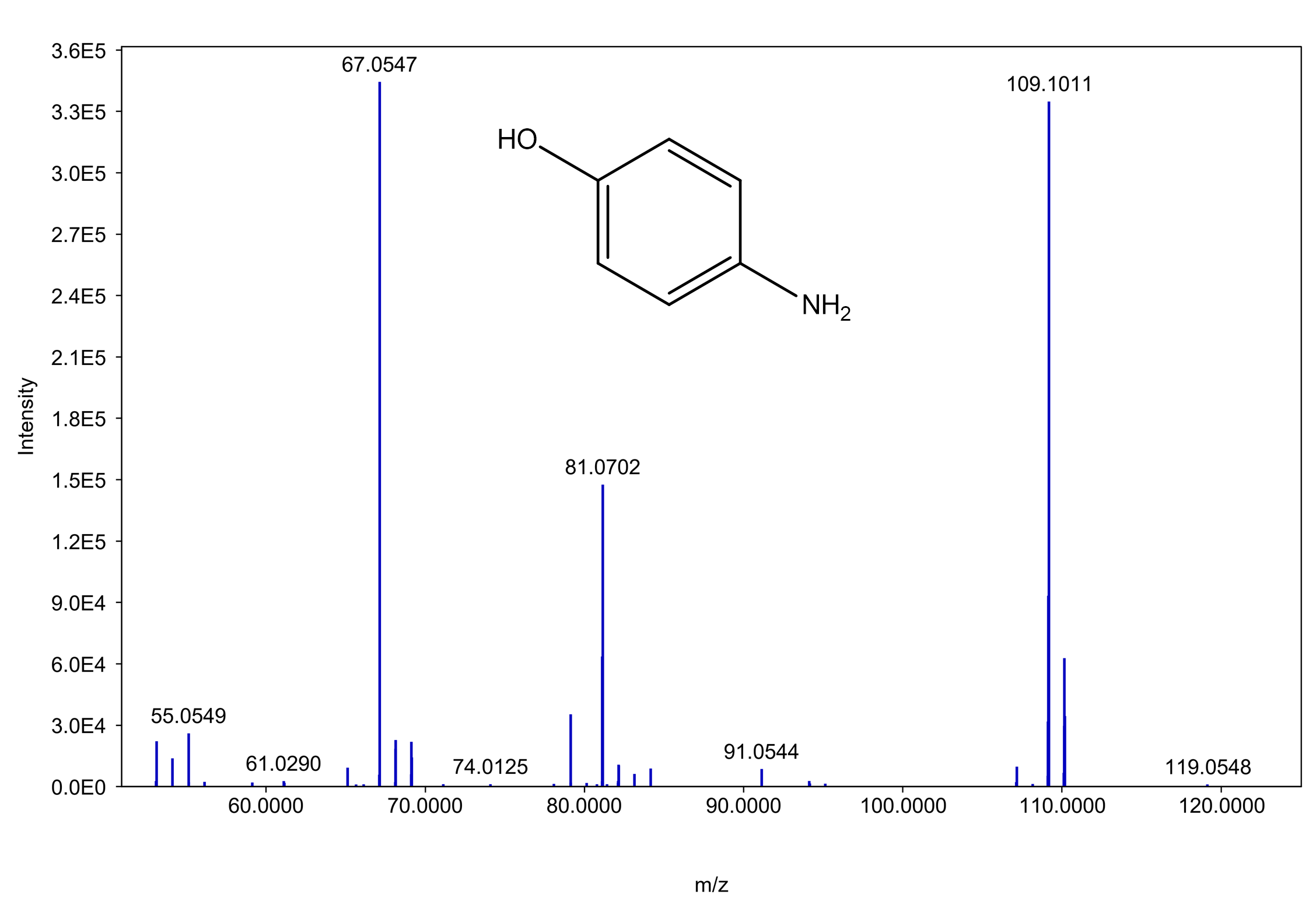 |
| Catechol | 2.3 | Chemical Formula: C6H6O2  Exact Mass: 110.04  Molecular Weight: 110.11  m/z: 110.04 (100.0%), 111.04 (6.6%) | 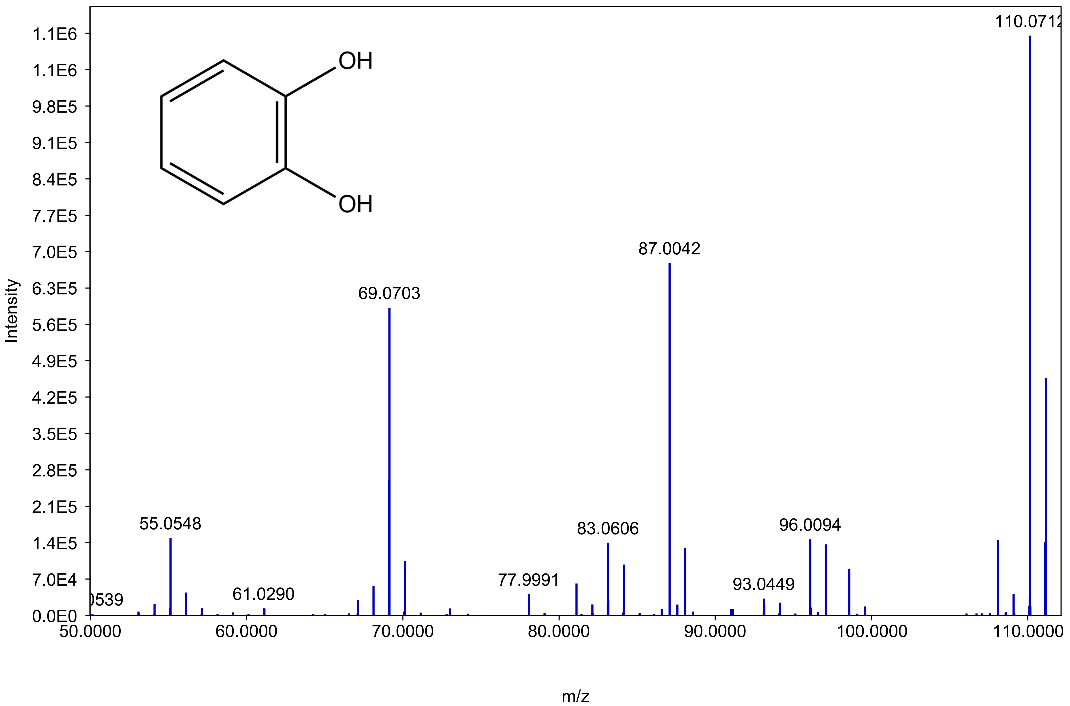 |
| Nitrobenzene | 2.33 | Chemical Formula: C6H5NO2  Exact Mass: 123.03  Molecular Weight: 123.11  m/z: 123.03 (100.0%), 124.04 (6.6%) | 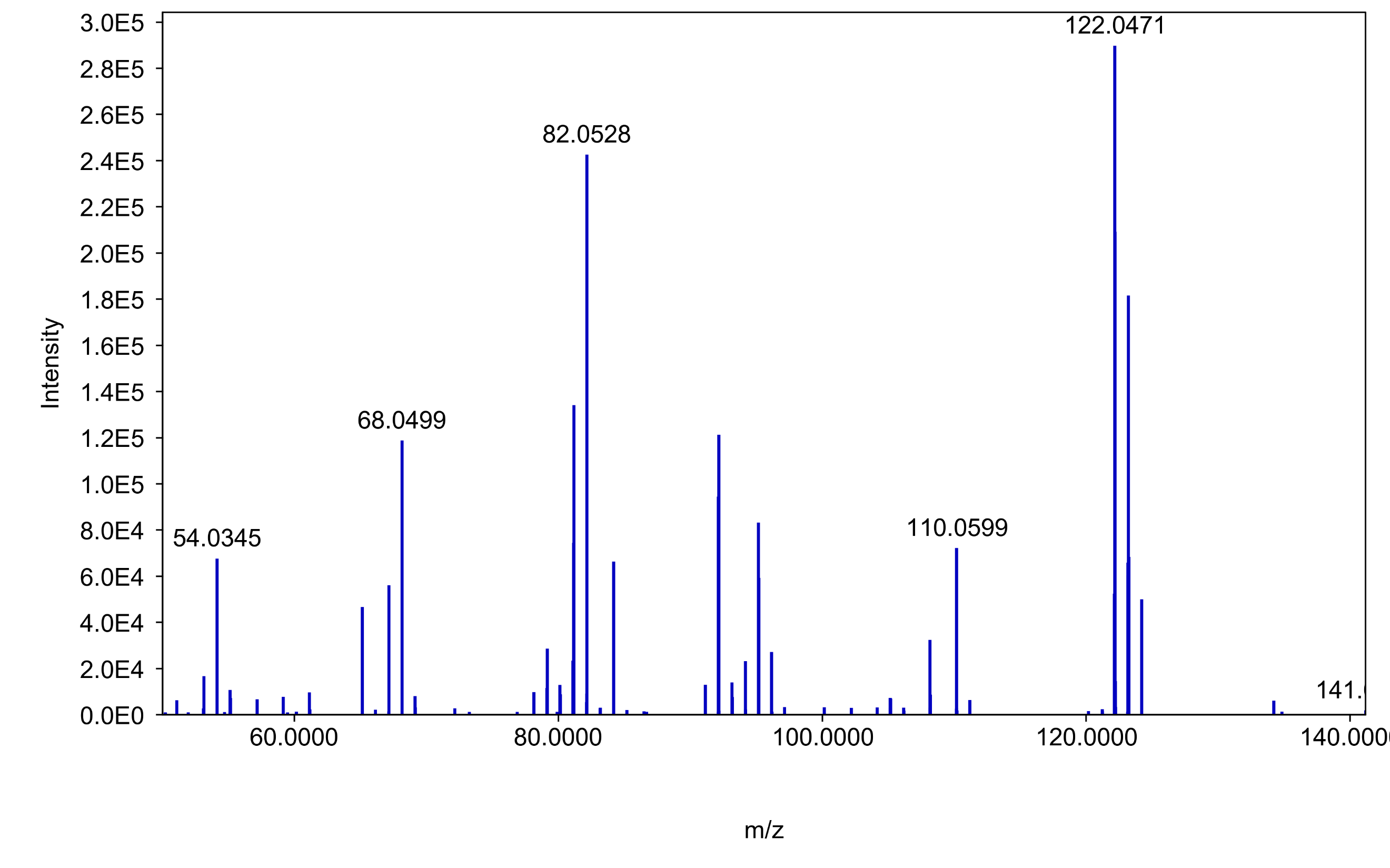 |
| 4-Nitrophenol | 2.36 | Chemical Formula: C6H5NO3  Exact Mass: 139.03  Molecular Weight: 139.11  m/z: 139.03 (100.0%), 140.03 (6.7%) | 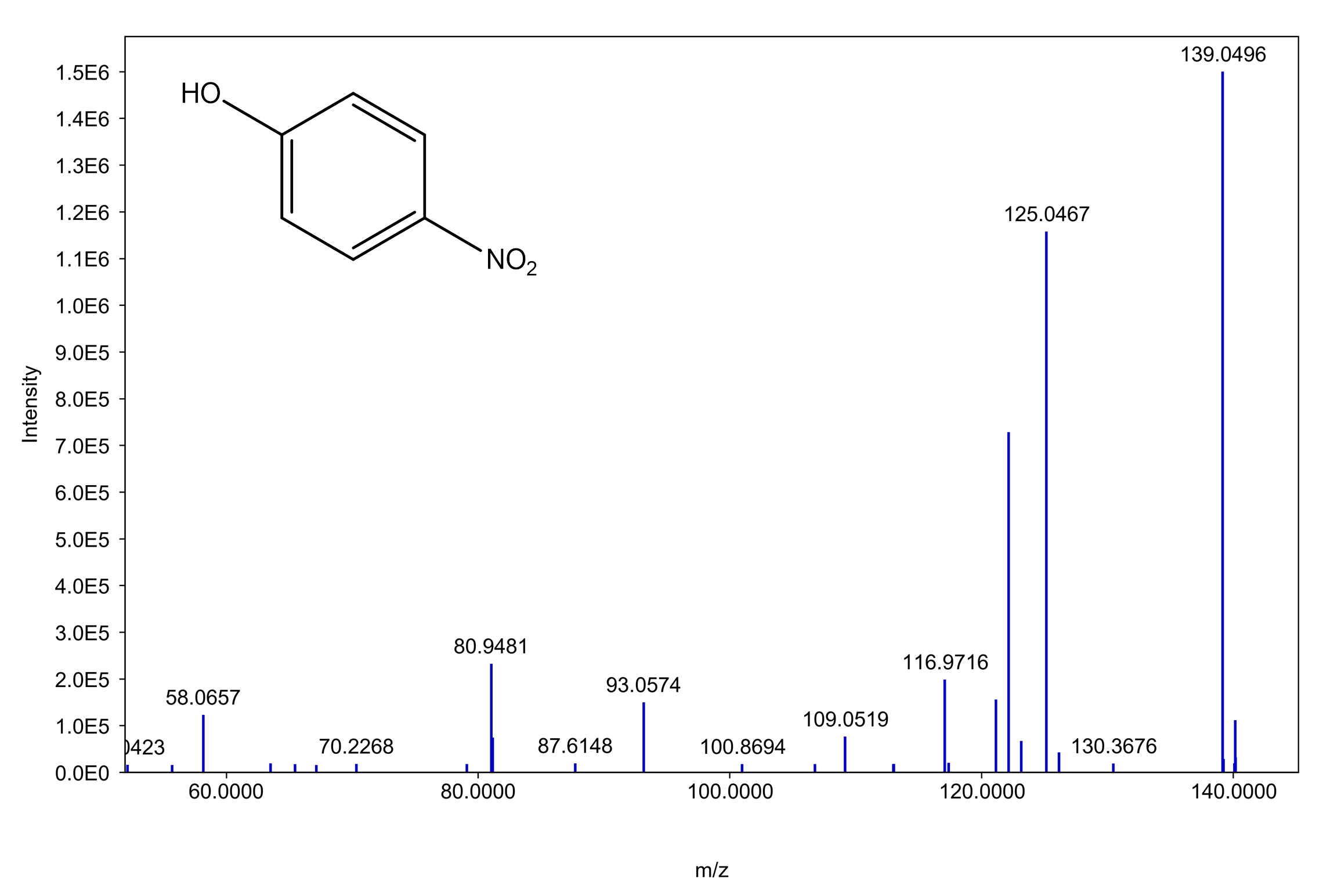 |
| Naphthalene-1,2,8-triol | 2.45 | Chemical Formula: C10H8O3  Exact Mass: 176.05  Molecular Weight: 176.17  m/z: 176.05 (100.0%), 177.05 (11.0%), 178.05 (1.2%) | 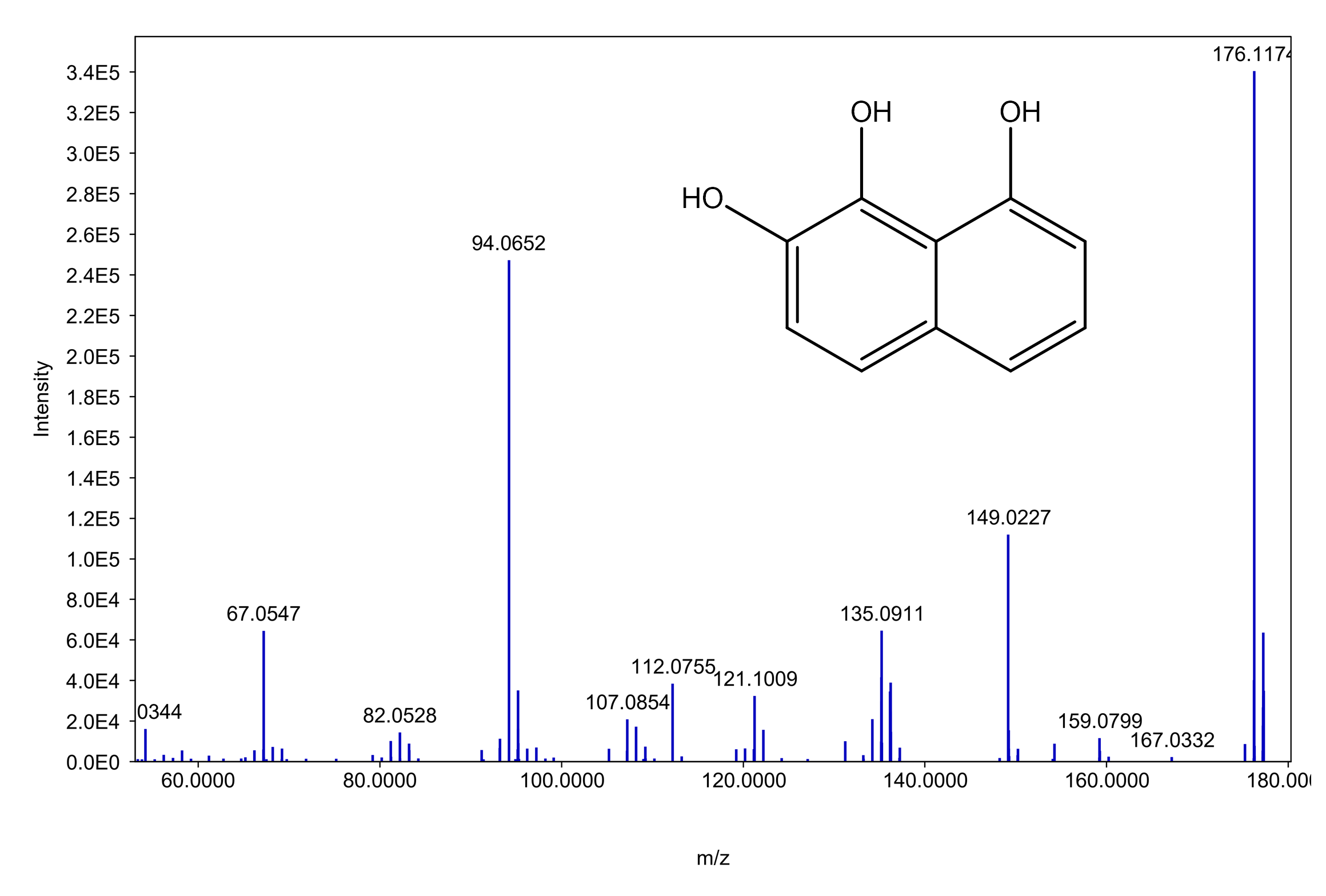 |
| 4-Nitroaniline | 2.7 | Chemical Formula: C6H6N2O2  Exact Mass: 138.04  Molecular Weight: 138.12  m/z: 138.04 (100.0%), 139.05 (6.6%) | 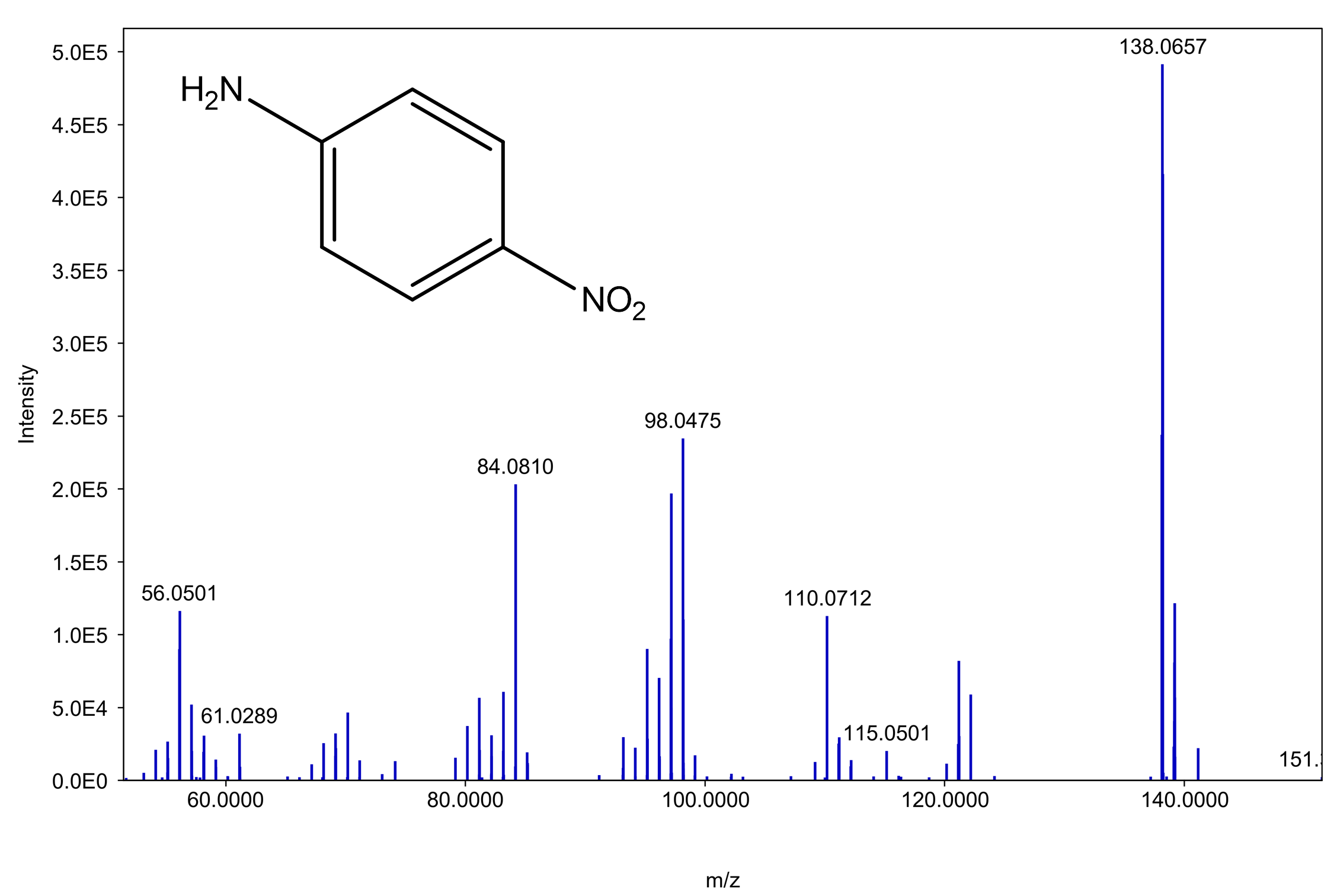 |
| (E)-2,8-diamino-7-((4-nitrophenyl)diazenyl) naphthalen-1-ol | 4.22 | Chemical Formula: C22H16N6O3  Exact Mass: 412.13  Molecular Weight: 412.40  m/z: 412.13 (100.0%), 413.13 (26.3%) | 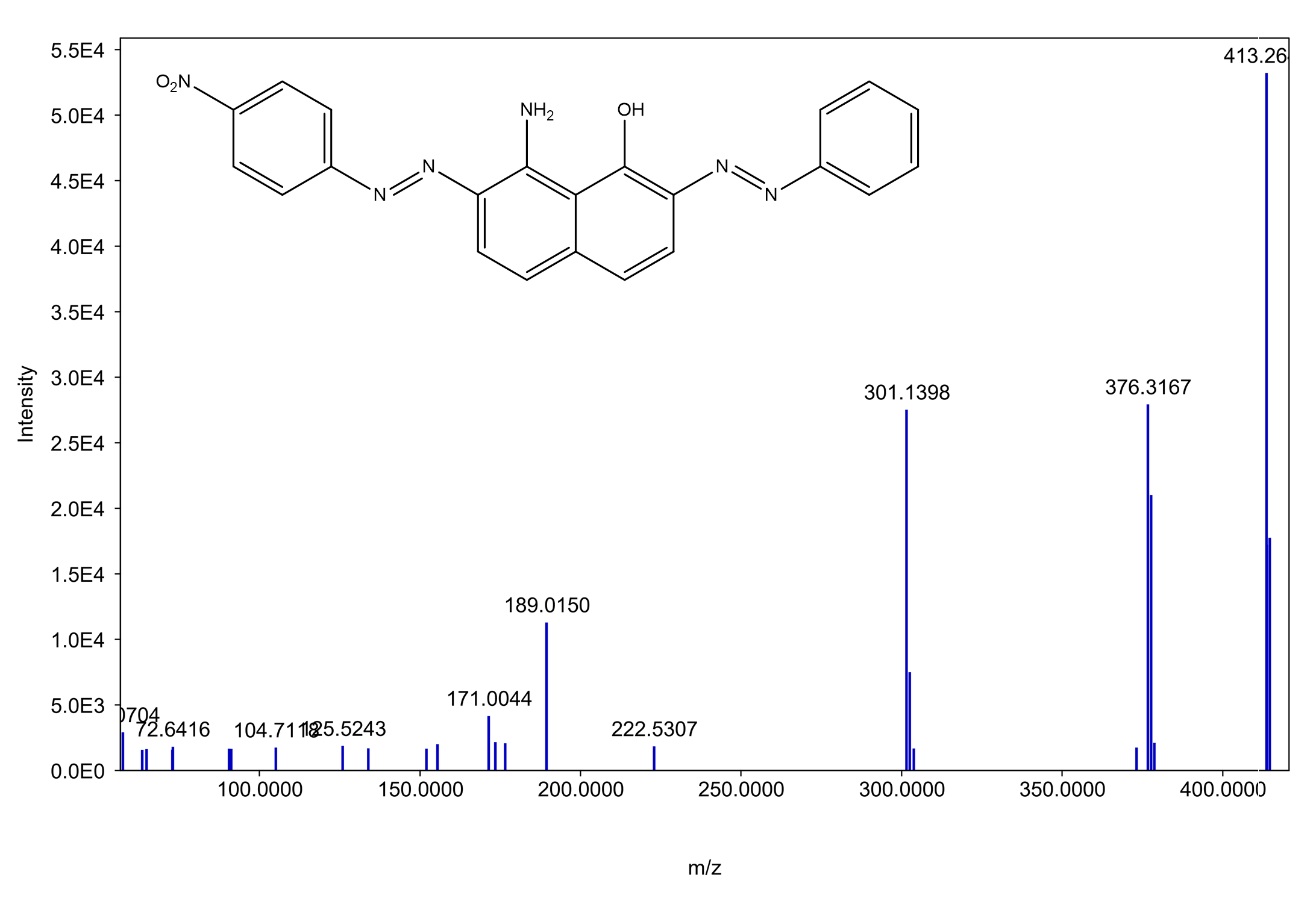 |

2. Supplementary Methods

**Method S1: Specifications of instrument for LCMS analysis of AB1 degradation products**

| **HPLC setting** | - Name of the instrument: Dionex Ultimate 3000, Thermo Scientific - Name of analytical column: Hypersil Gold, Thermo Scientific (5 µm ,100cm ×2.1µm) - Solvent System: Acetonitrile:water(70:30) |
| --- | --- |
| **Mass Spectrometer settings** | - Name of the instrument: Q Exactive, Thermo Scientific - Run Time: 0 to 20 min - Polarity: Positive - Default charge state: 1 - ***Full MS setting:***   Microscan: 1  Resolution: 70000  AGC (Automatic gain control): 1e6  Maximum IT (ion transfer) time: 100 ms  Scan range: 100 to 1200 m/z   - ***(MS2) setting:***   Microscan: 1  Resolution: 35000  AGC (Automatic gain control): 1e5  Maximum IT (ion transfer): 50 ms  Loop count: 5  Top N: 5  Isolation window: 4.0 m/z  Isolation offset: 1.0 m/z   - Fixed first mass: - - Normalized collision energy: 30 - Data dependent settings - Minimum AGC target: 7.50 e3 - Charge exclusion: unassigned - Dynamic exclusion time: 8.0 S |
| **Compound discoverer settings** | - Version: Compound Discoverer 2.1.0.401 - Mass Tolerance [ppm]: 5 ppm - Intensity Tolerance [%]: 30 - S/N Threshold: 5 - Min. Peak Intensity: 500000 - Database: m/z cloud and chemspider. |

3. Supplementary Figures


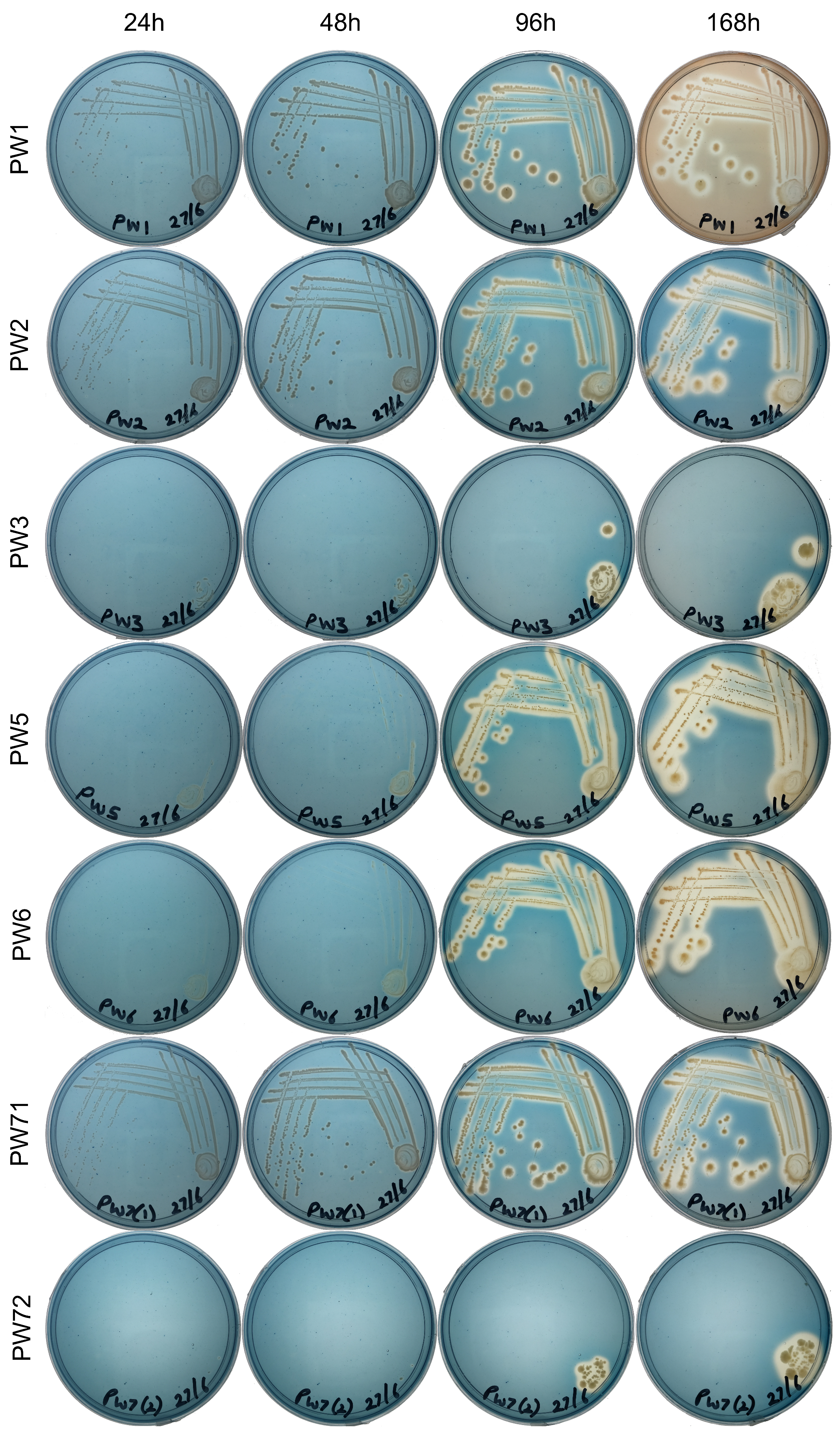


**Supplementary Fig. S1** **The results from the CAS agar plate assay suggest the siderophore production in all 7 bacterial isolates though with varying growth in the iron limiting MM9 media.** The siderophore production was confirmed as the appearance of the orange-yellow zone due to siderophore-mediated removal of Fe from blue-colored CAS-HDTMA-Fe complex. The strains *Serratia plymuthica* PW1, *Ralstonia pickettii* PW2, and *Serratia liquefaciens* PW71 showed higher growth as the bacterial colonies appeared after 24 h of streaking. The bacterial isolates *Stenotrophomonas maltophilia* PW5 and *Stenotrophomonas maltophilia* PW6, though did not show immediate growth in the deferrated MM9 media, produced siderophore after 96 h of streaking. The bacterial strains *Stenotrophomonas rhizophila* PW3 and *Stenotrophomonas rhizophila* PW72 showed limited growth in MM9 media.


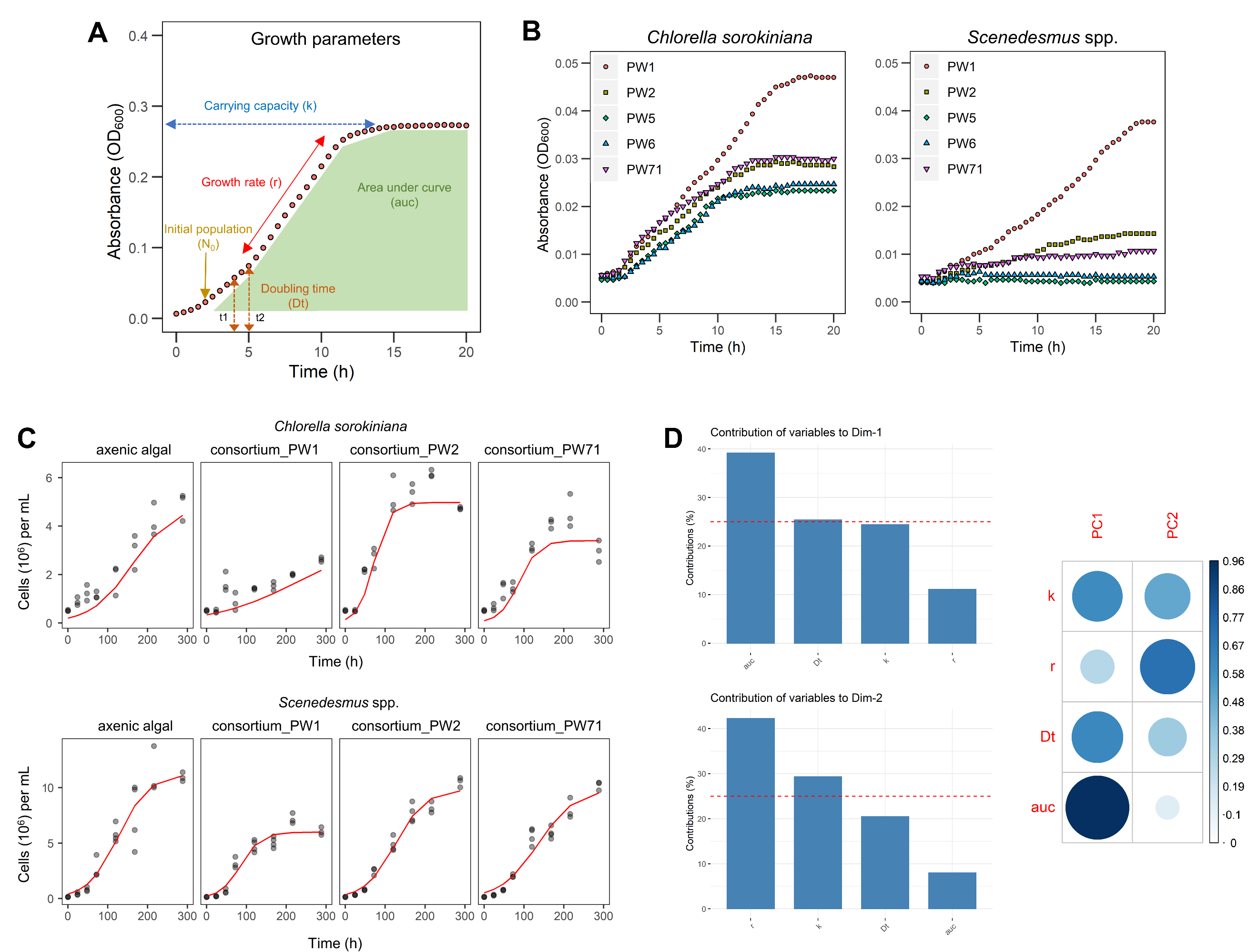


**Supplementary Fig. S2** **Different growth parameters obtained from fitting the growth data using package ‘*growthcurver*’ in R. A** The logistic equation Nt=k/(1+(k−N0/N0)e−rt) are explained as follows: the growth rate (r) represents an increase in the population of bacterial strains (per unit time) during the exponential growth phase. The doubling time (Dt) represents the time required for the microbial population to double in size (cell count) during the log phase. The carrying capacity (k) represents the maximum population size in the system, and area under curve (auc) is an integrative metric computed using the carrying capacity (k), growth rate (r), and initial population size (N_0_) which represents the total area under the trapezoid-shaped growth curve. **B** Bacterial growth curves performed on exudates of *C. sorokiniana* and *Scenedesmus* sp. showed bacteria prefer exudates of *C. sorokiniana*. **C** The prediction model (red line) computed by fitting the observed algal growth data (grey dots) in the logistic equation using the prediction modelling package ‘*growthcurver*’ in R [9]. **D** The percent contribution of growth variables in PC1 and PC2 after the PCA analysis.


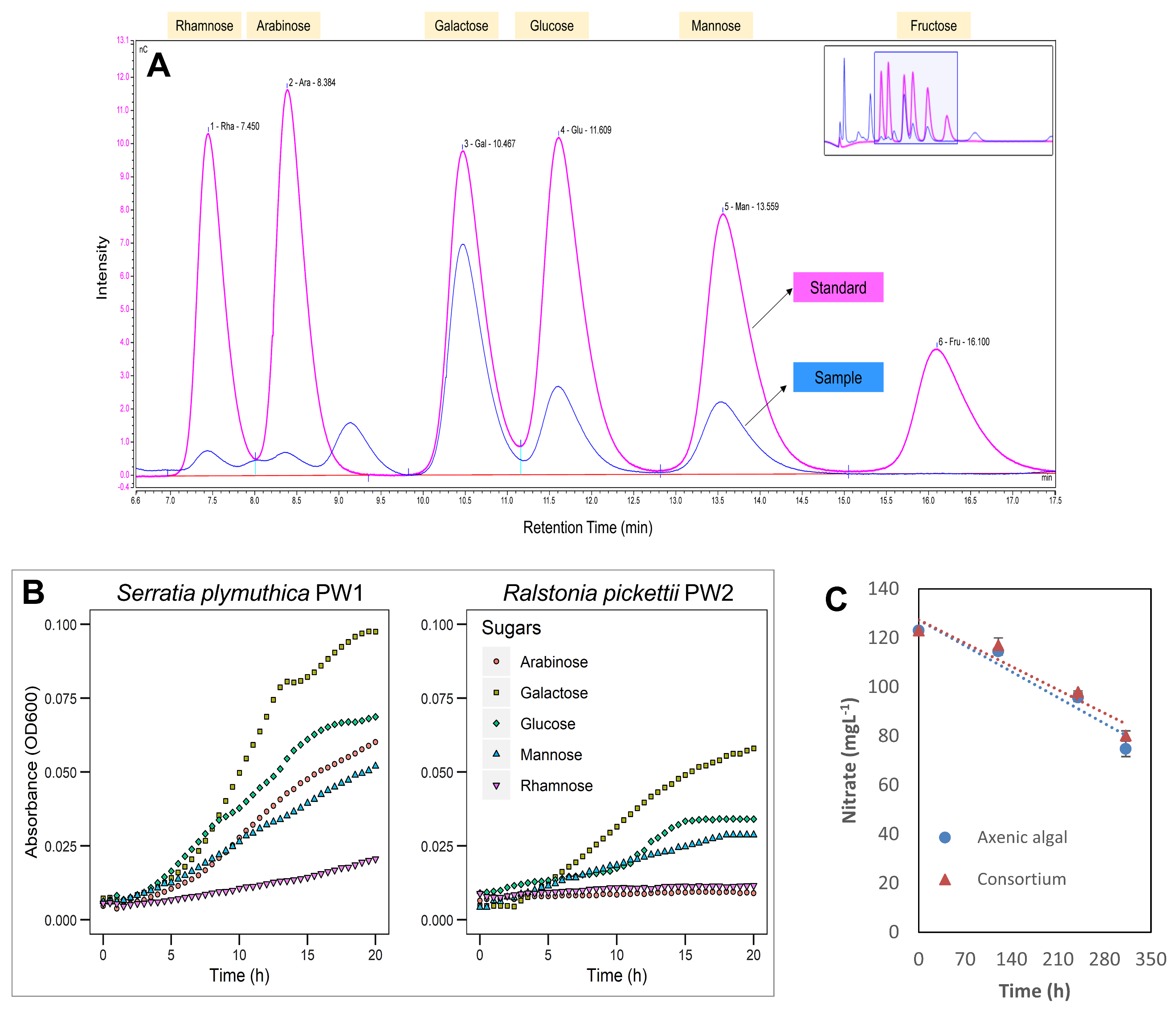
 **Supplementary Fig. S3 A** Results of the anion-exchange chromatography and growth of bacterial strains in different carbon sources**.** The chromatogram after high-performance anion-exchange chromatography represents the identification rhamnose, arabinose, galactose, glucose, and mannose in the EPS extracted from *Chlorella sorokiniana*. **B** The bacterial growth curves in different sugars suggest high culturability of S. plymuthica PW1 in all 5 sugars, whereas, R. pickettii PW2 shows limited culturability only in galactose followed by glucose and mannose. **C** Nitrate concentrations over the period of 312 hours of growth in axenic algal and consortium setups of *Chlorella sorokiniana*.


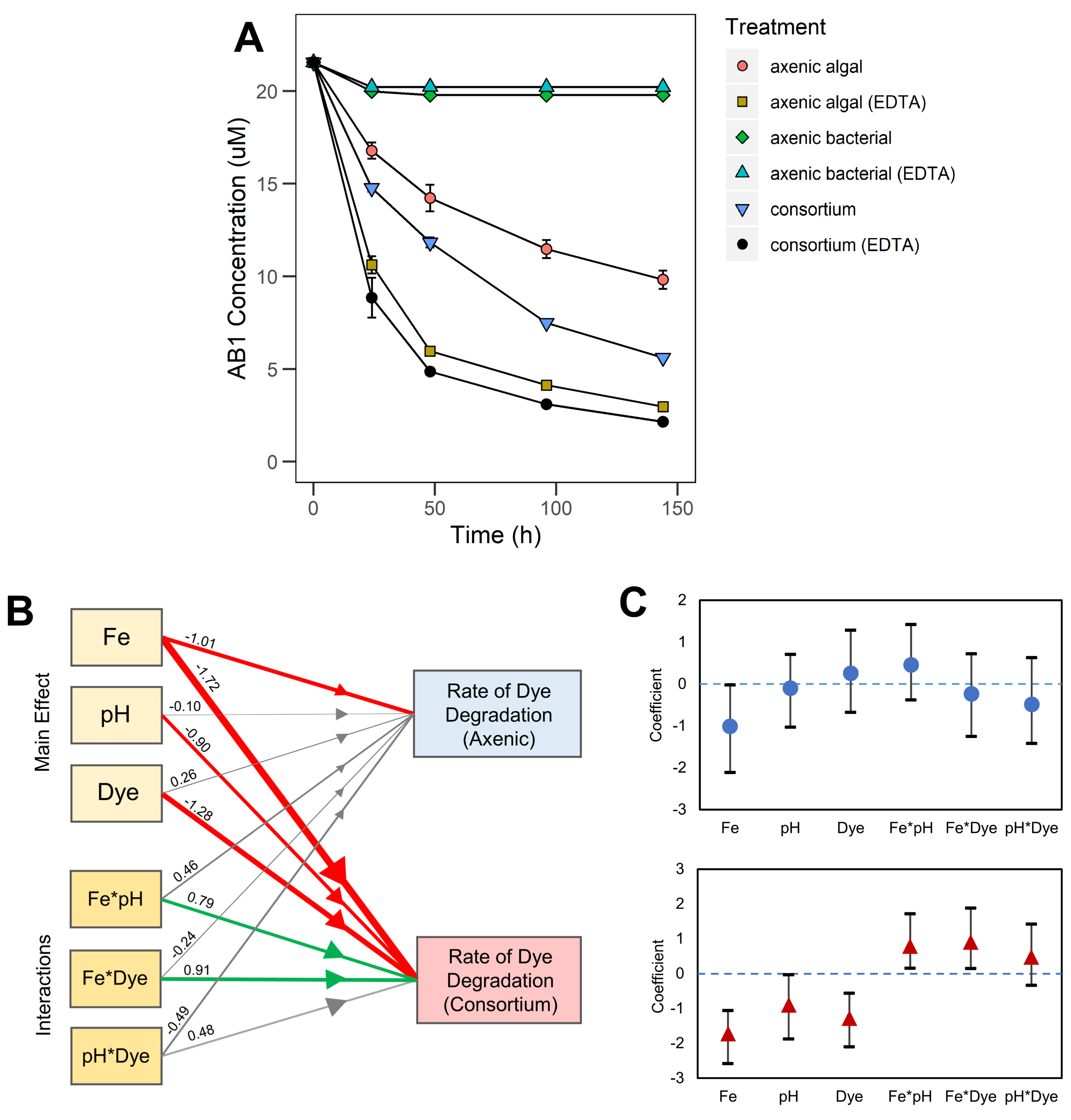


**Supplementary Fig. S4** **The Acid Black 1 dye degradation under varied condition of iron bioavailability suggests a higher dye degradation in treatment setups with EDTA supplemented iron.** **A** The bacterial strain *Ralstonia pickettii* PW2 enhanced the algal dye degradation under culture conditions where iron was supplemented without EDTA (consortium). **B** Output of the Partial Least Squares Path (PLSP) modeling of multifactorial L_16_ design. The red and green line represents significant effect of the change in factors and their interactions on the rate of dye degradation in axenic and consortium setups. Grey line represents non-significant impact. The thickness of lines represents the values of path coefficients. **C** The confidence interval plot of the output of the Partial Least Squares Path (PLSP) modeling of multifactorial L_16_ design.


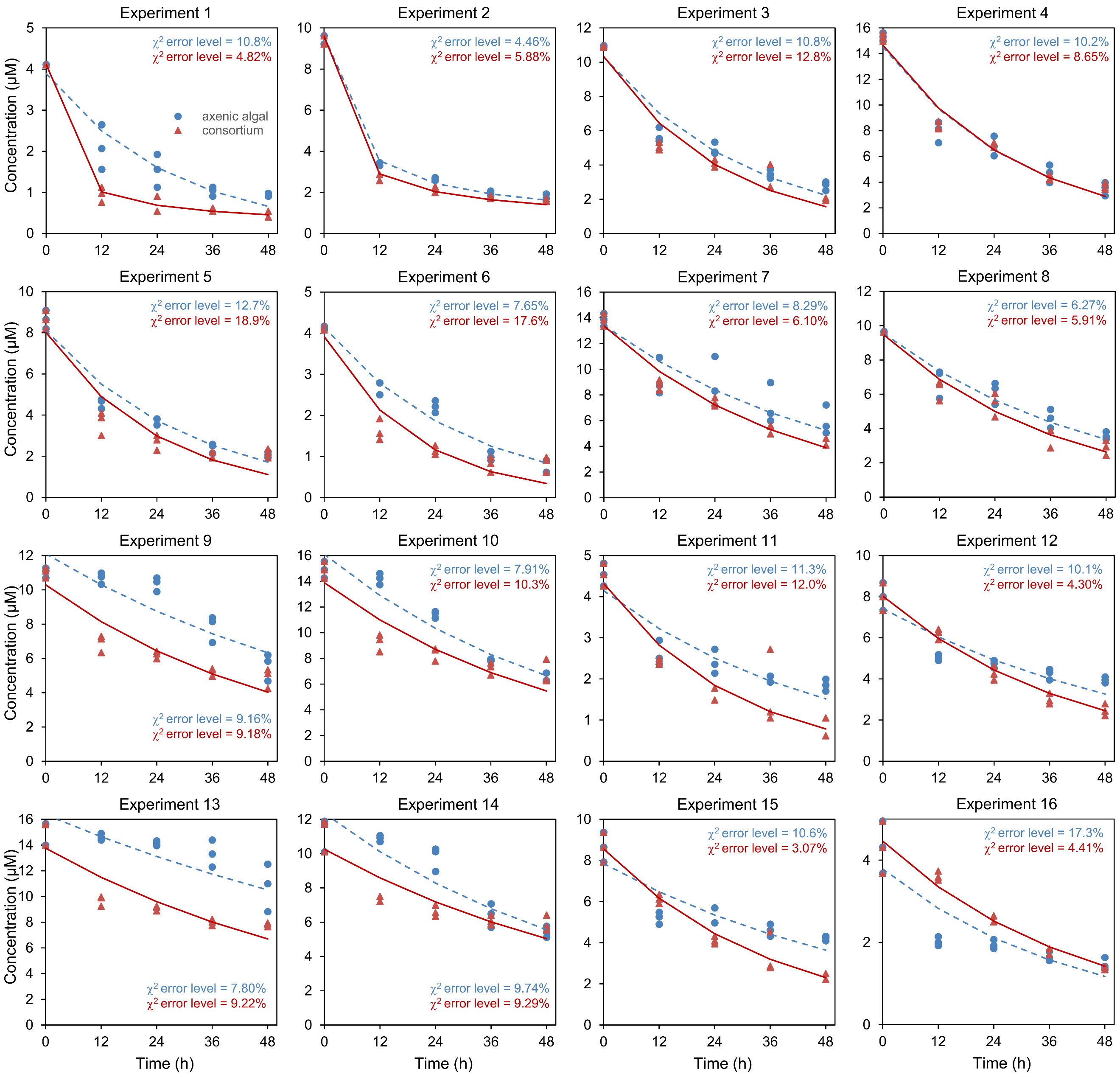


**Supplementary Fig. S5** Degradation of Acid Black 1 (AB1) dye under different experimental conditions of L_16_ orthogonal array design (Experiment 1-16) in axenic algal alone (dotted blue lines) and algal-bacterial consortium (solid red lines) setups over a period of 48 hours.


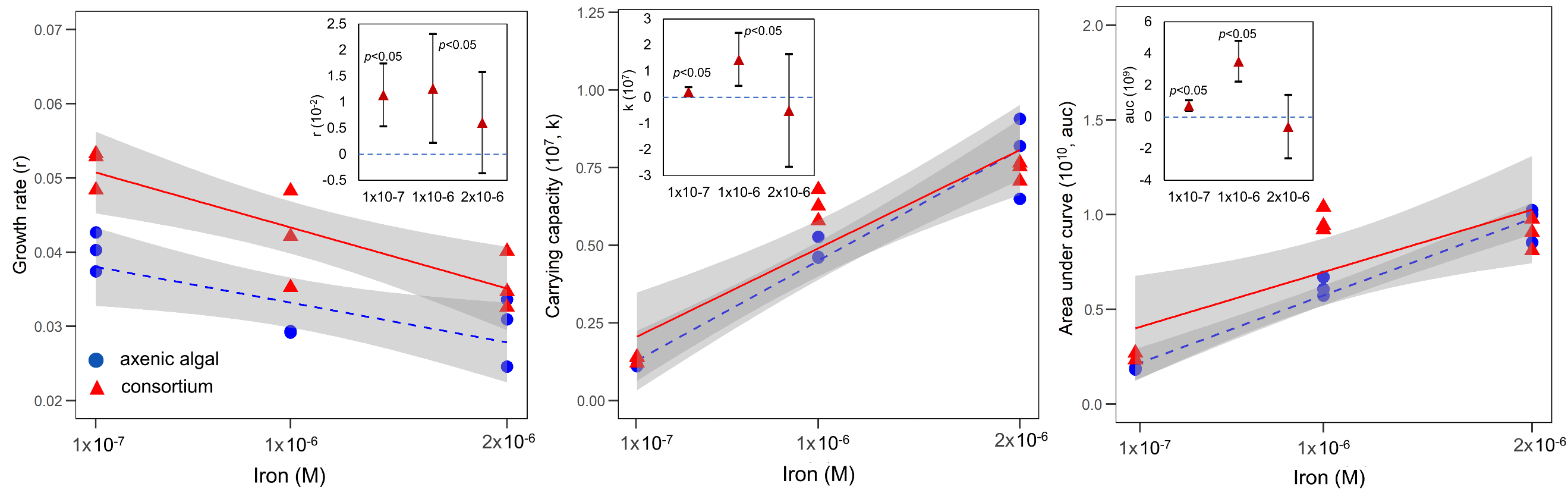


**Supplementary Fig. S6** The confidence interval plots of different growth parameters of algal growth in consortium w.r.t. axenic growth (horizontal blue dashed line) suggests growth promotional properties of *Ralstonia pickettii* PW2 under low concentration of iron. The bacterium significantly enhanced the growth rate, carrying capacity, and area under curve of *Chlorella sorokiniana* at Fe concentration of 1x10^-7^ and 1x10^-6^ M. On the contrary, the effect of the presence of bacteria at a higher Fe concentration of 2x10^-6^ M was not significant (inset).


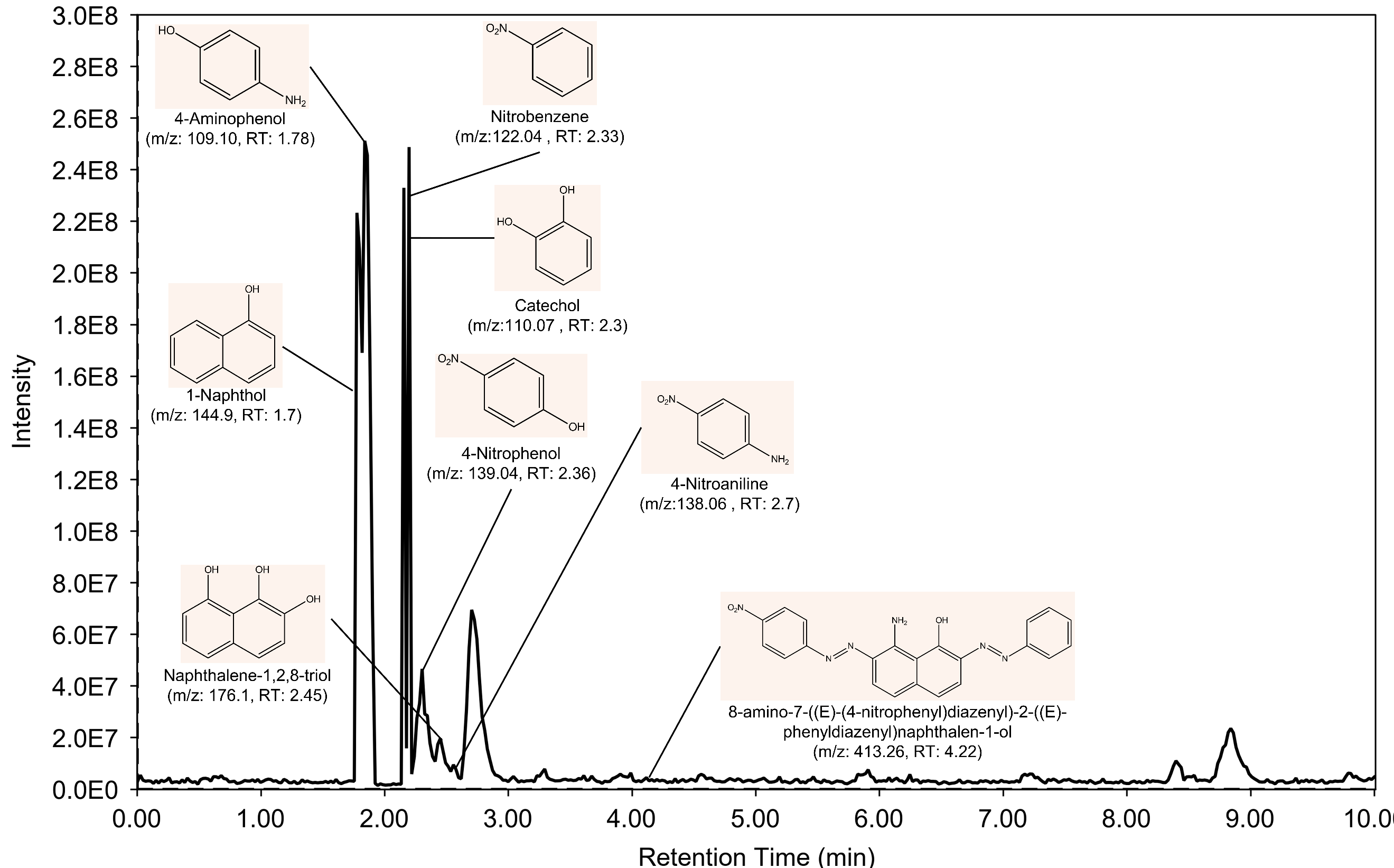


**Supplementary Fig. S7** The chromatograms of AB1 biodegraded products after LC-MS analysis.


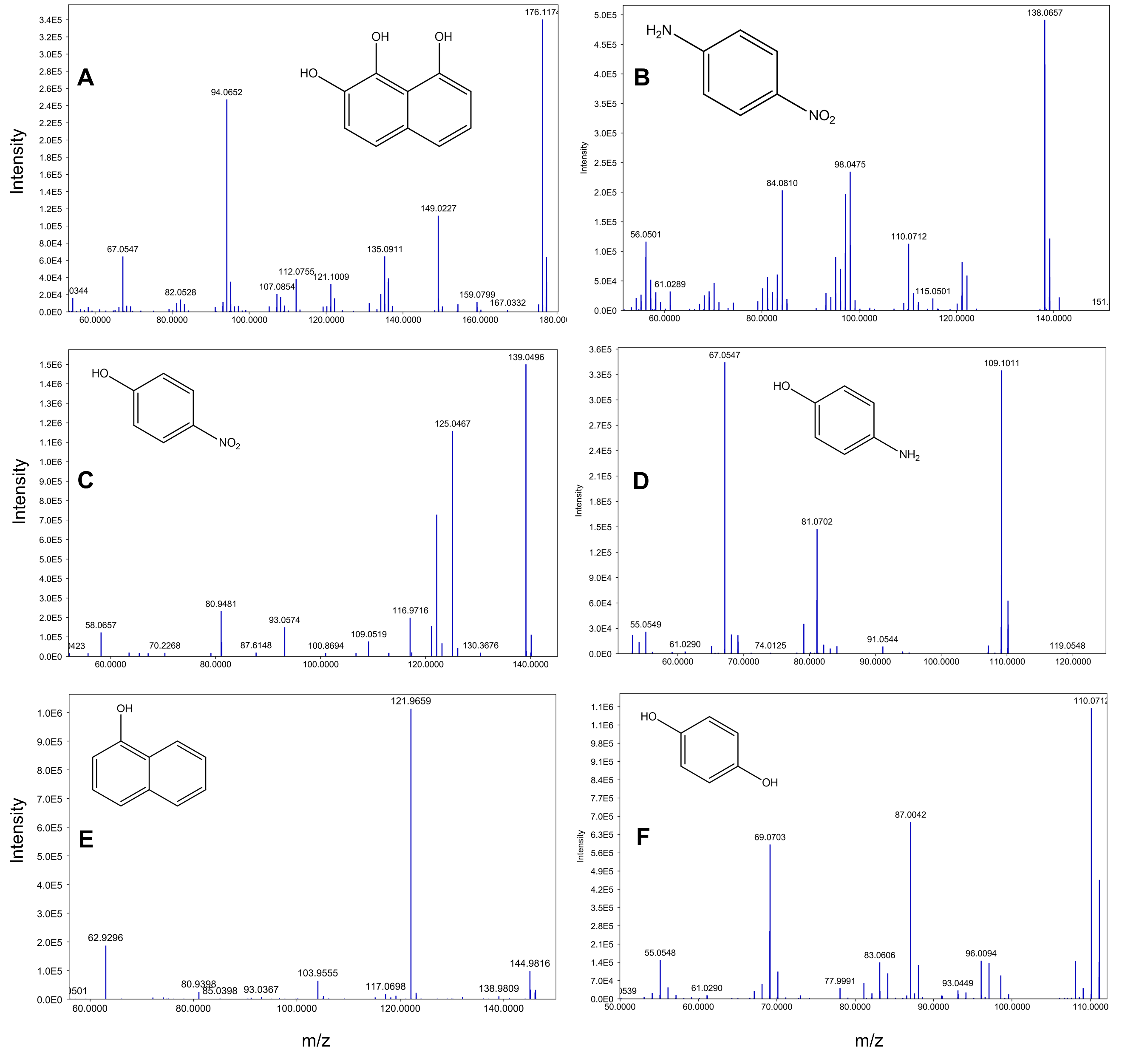


**Supplementary Fig. S8** Mass spectra of ABI biodegraded products showing formation of **A** Naphthalene-1,2,8-triol, **B** 4-Nitroaniline, **C** 4-Nitrophenol, **D** 4-Aminophenol, **E** Naphthalene-1-ol, and **F** Catechol.


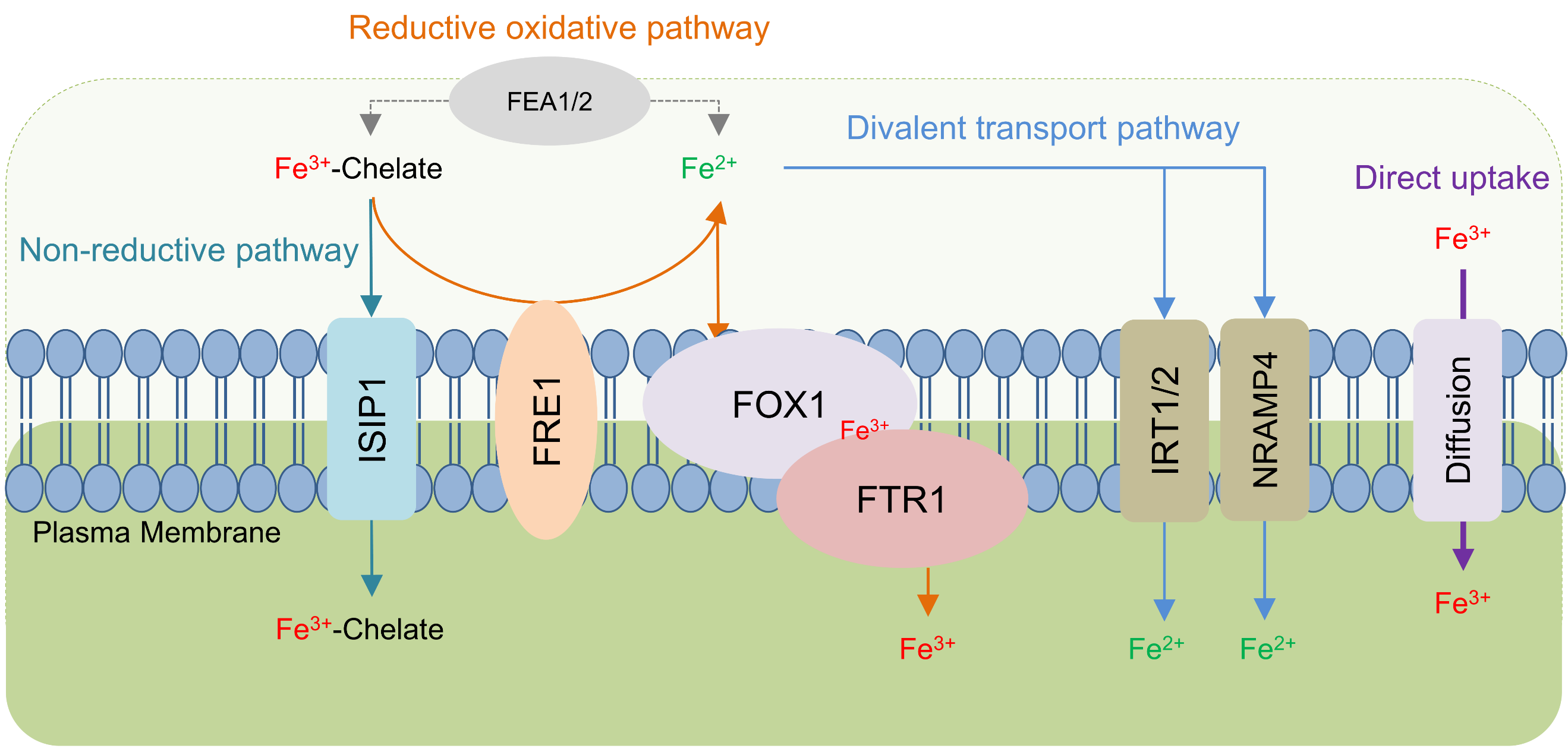


**Supplementary Fig. S9** Different Fe-uptake mechanisms, as observed in various single cell eukaryotic organisms, can be divided into the reductive oxidative pathway, divalent transport pathway, non-reductive uptake, and direct diffusion (check Supplementary Table 14 for more information). The reductive oxidative pathway (ferrireductase pathway) reported in dinoflagellate *Scrippsiella trochoidea* [13], marine algae *Phaeodactylum tricornutum* [21, 22], and freshwater alga *Chlamydomonas reinhardtii* [23], begins with the cell surface reduction of chelated Fe^3+^ to Fe^2+^ by a plasma membrane-bound NADPH-oxidoreductases (NOX family) FRE1 protein [24]. This iron uptake pathways has been discussed in detail in model eukaryote *Saccharomyces cerevisiae* (baker’s yeast) [21]. The extracellular FEA1 and FEA2 protein here plays a vital role in the accumulation of Fe-chelates near the cell surface of *Chlamydomonas reinhardtii* as they bind with the ferric (Fe^3+^) iron and make it available for reduction by FRE1 [23]. The second stage includes the further oxidation of ferrous (Fe^2+^) iron by a multicopper ferroxidase (FOX1) and the transfer of Fe^3+^ to a transporter ferric permease protein (FTR1) as observed in freshwater alga *Chlamydomonas reinhardtii*. Allen et al., 2007 also postulated that FEA1/2 may also play a vital role in increasing the bioavailability of Fe^2+^ for FOX1 as the proteins have been known to increase the solubility of ferrous iron. On the other hand, in a divalent transport pathway, the reduced Fe^2+^ is directly incorporated into the cell via zip (ZIP) family proteins (IRT1 and IRT2) as discussed in model green alga *Ostreococcus tauri* [25] and natural resistance-associated macrophage proteins (NRAMP4) discussed in *Chlamydomonas reinhardtii* [26]. The zip family proteins have been known to transport divalent ions like zinc and iron into the cell. The direct incorporation of Fe-siderophore chelate has also been reported in an endocytosis-mediated uptake by iron starvation-induced protein 1 (ISIP1), which is a non-reductive iron uptake pathway as reported in *Phaeodactylum tricornutum* [27]. Also, at higher iron concentration, it has been reported than ferric iron can diffuse directly via cell membrane, and gets further reduced inside the cell as observed in photosynthetic marine alveolate *Chromera velia* [28, 29].


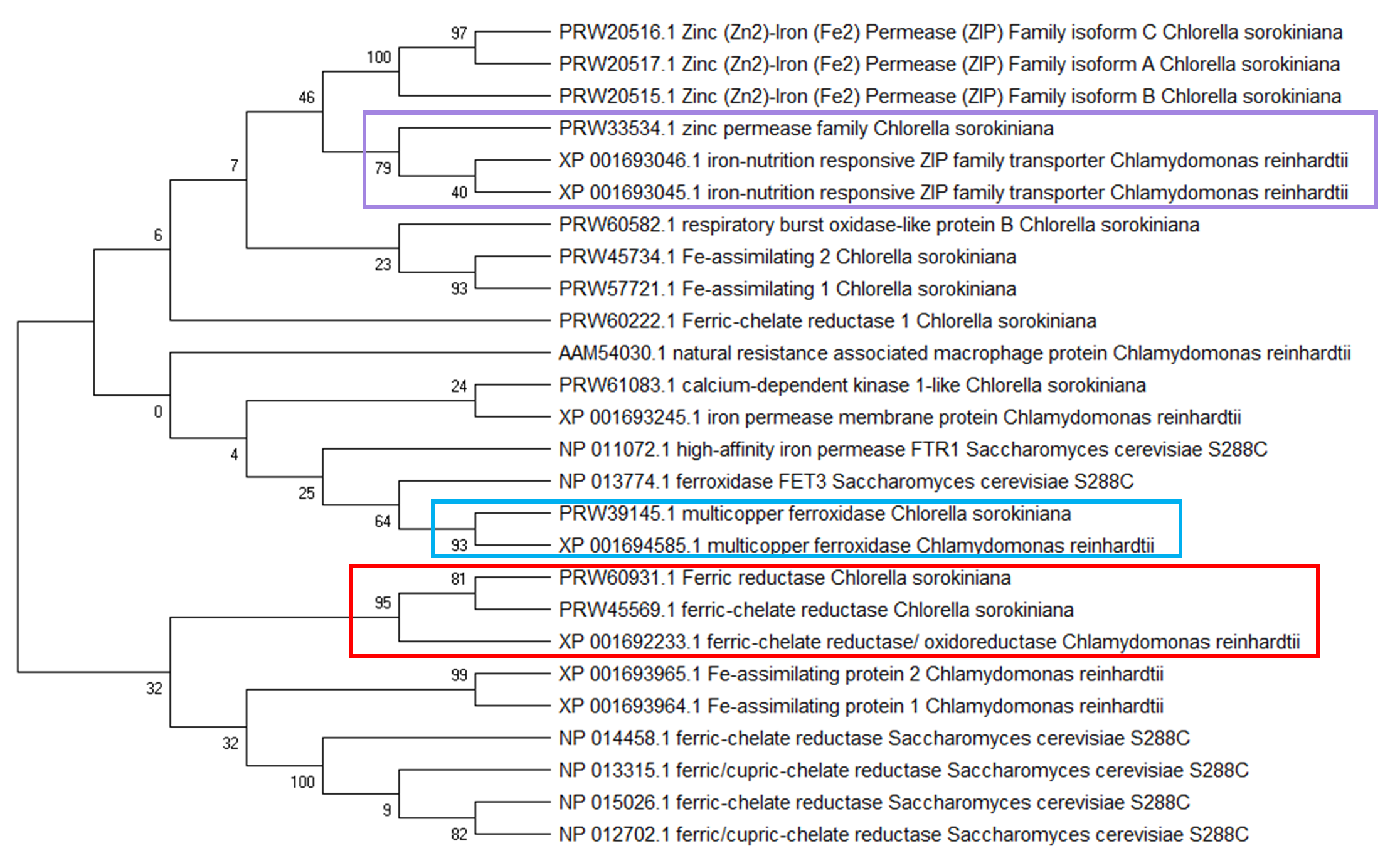


**Supplementary Fig. S10** The evolutionary history was inferred by using the Maximum Likelihood method and JTT matrix-based model in MEGA X software. The percentage of trees in which the associated taxa clustered together is shown next to the branches (a total of 500 bootstrap replication were used). This analysis involved 26 amino acid sequences from species *Chlorella sorokinana* UTEX 1602, *Chlamydomonas reinhardtii* and *Saccharomyces cerevisiae*. The protein sequences with maximum likelihood and higher bootstrap value are highlighted using color codes. The red color indicates the FRE1 (ferric- chelate reductase like) proteins showing a close relation between *Chlorella sorokiniana* and *Chlamydomonas reinhardtii*.

4. Supplementary Tables

Supplementary Table S1: The difference in the growth parameters of the microalgae *Chlorella sorokiniana* and *Scenedesmus* spp. co-cultured in the presence of siderophore producing bacteria.

| **Growth parameter** | **Co-culture treatments** | **Algae** | | **t-test (*p* value)** |
| --- | --- | --- | --- | --- |
|  |  | *Chlorella sorokiniana* | *Scenedesmus* spp. |  |
| Carrying capacity (k) | axenic algal | 4.82±0.45 x10^6^ | 11.25±0.83 x10^6^ | 0.001 |
|  | *Serratia plymuthica* PW1 | 3.34±1.99 x10^6^ | 5.99±0.27 x10^6^ | 0.000 |
|  | *Ralstonia pickettii* PW2 | 4.97±0.13 x10^6^ | 9.85±0.38 x10^6^ | 0.001 |
|  | *Serratia liquefaciens* PW71 | 3.40±0.21 x10^6^ | 9.88±0.66 x10^6^ | 0.003 |
| Growth rate (r) | axenic algal | 1.95±0.33 x10^-2^ | 2.60±0.51 x10^-2^ | 0.080 |
|  | *Serratia plymuthica* PW1 | 0.97±0.41 x10^-2^ | 3.89±0.74 x10^-2^ | 0.081 |
|  | *Ralstonia pickettii* PW2 | 5.02±1.09 x10^-2^ | 2.63±0.27 x10^-2^ | 0.006 |
|  | *Serratia liquefaciens* PW71 | 4.14±1.13 x10^-2^ | 2.14±0.31 x10^-2^ | 0.002 |
| Doubling time (Dt) | axenic algal | 35.45±3.905 | 26.65±5.337 | 0.078 |
|  | *Serratia plymuthica* PW1 | 70.98±10.29 | 17.79±20.78 | 0.010 |
|  | *Ralstonia pickettii* PW2 | 13.80±1.060 | 26.28±5.641 | 0.007 |
|  | *Serratia liquefaciens* PW71 | 16.71±0.4562 | 32.36±6.411 | 0.023 |
| Area under curve (auc) | axenic algal | 6.01±0.49 x10^8^ | 18.05±4.73 x10^8^ | 0.005 |
|  | *Serratia plymuthica* PW1 | 3.26±1.51 x10^8^ | 12.13±3.08 x10^8^ | 0.000 |
|  | *Ralstonia pickettii* PW2 | 11.01±0.37 x10^8^ | 15.83±1.25 x10^8^ | 0.002 |
|  | *Serratia liquefaciens* PW71 | 7.05±0.25 x10^8^ | 15.07±2.40 x10^8^ | 0.000 |

Supplementary Table S2: The output of Levene’s, One-way ANOVA, and Tukey’s posthoc tests used to compute the difference in the growth parameters of *Chlorella sorokiniana* and *Scenedesmus* spp. algae co-cultured in the presence of siderophore producing bacterial strains.

| **Growth parameters** | **Algae** | **Levene** | | **ANOVA** | | | **Tukey** | |
| --- | --- | --- | --- | --- | --- | --- | --- | --- |
|  |  | **Levene Statistic** | ***p* value** | **Sum of squares** | **F statistics** | ***p* value** | **Association of axenic algal w.r.t** | ***p* value** |
| Growth rate (r) | *Chlorella sorokiniana* | 2.495 | 0.134 | 0.003 (Between groups)  0.000 (within groups) | 51.5 | 0.000 | consortium_PW1 | 0.068 |
|  |  |  |  |  |  |  | consortium_PW2 | 0.000 |
|  |  |  |  |  |  |  | consortium_PW71 | 0.002 |
|  | *Scenedesmus* spp. | 7.858 | 0.009 | 0.005 (Between groups)  0.005 (within groups) | 11.731 | 2.632 | consortium_PW1 | 0.261 |
|  |  |  |  |  |  |  | consortium_PW2 | 0.989 |
|  |  |  |  |  |  |  | consortium_PW71 | 0.968 |
| Carrying capacity (k) | *Chlorella sorokiniana* | 1.707 | 0.242 | 8.77x10^12^ (Between groups)  3.50x10^12^ (within groups) | 6.674 | 0.014 | consortium_PW1 | 0.004 |
|  |  |  |  |  |  |  | consortium_PW2 | 1.000 |
|  |  |  |  |  |  |  | consortium_PW71 | 0.067 |
|  | *Scenedesmus* spp. | 3.726 | 0.061 | 4.91x10^13^ (Between groups)  4.80x10^12^ (within groups) | 27.273 | 0.000 | consortium_PW1 | 0.000 |
|  |  |  |  |  |  |  | consortium_PW2 | 0.231 |
|  |  |  |  |  |  |  | consortium_PW71 | 0.263 |
| Doubling time (Dt) | *Chlorella sorokiniana* | 4.468 | 0.04 | 5690.06 (Between groups)  737.36 (within groups) | 10.362 | 20.578 | consortium_PW1 | 0.009 |
|  |  |  |  |  |  |  | consortium_PW2 | 0.145 |
|  |  |  |  |  |  |  | consortium_PW71 | 0.218 |
|  | *Scenedesmus* spp. | 0.352 | 0.789 | 623.2 (Between groups)  325.73 (within groups) | 5.102 | 0.029 | consortium_PW1 | 0.261 |
|  |  |  |  |  |  |  | consortium_PW2 | 0.788 |
|  |  |  |  |  |  |  | consortium_PW71 | 0.391 |
| Area under curve (auc) | *Chlorella sorokiniana* | 2.564 | 0.128 | 8.92x10^17^ (Between groups)  2.75x10^16^ (within groups) | 86.391 | 0.000 | consortium_PW1 | 0.001 |
|  |  |  |  |  |  |  | consortium_PW2 | 0.000 |
|  |  |  |  |  |  |  | consortium_PW71 | 0.430 |
|  | *Scenedesmus* spp. | 4.66 | 0.036 | 6.24x10^17^ (Between groups)  1.75x10^17^ (within groups) | 9.513 | 0.005 | consortium_PW1 | 0.003 |
|  |  |  |  |  |  |  | consortium_PW2 | 0.086 |
|  |  |  |  |  |  |  | consortium_PW71 | 0.033 |

Supplementary Table S3: The output of the AB1 degradation kinetic modeling performed using ‘*mkin*’ package in R.

| **Treatment setup** | **Kinetic model** | **Chi-square Error (%)** | **Half-life  (h)** | **Degradation after 144 h (%)** |
| --- | --- | --- | --- | --- |
| axenic algal | SFO | 6.015 | 0.005 ± 0.0004 | 54.40 ± 2.28 |
| consortium | SFO | 8.557 | 0.009 ± 0.0000 | 73.96 ± 0.28 |
| axenic bacterial | NA | NA | NA | 8.15 ± 0.32 |
| axenic algal (EDTA) | FOMC | 4.202 | 0.033 ± 0.0027 | 86.21 ± 0.85 |
| consortium (EDTA) | FOMC | 2.859 | 0.049 ± 0.0078 | 89.99 ± 0.05 |
| axenic bacterial (EDTA) | NA | NA | NA | 6.16 ± 0.24 |

Supplementary Table S4: The output of Levene’s, One-way ANOVA, and Tukey’s posthoc tests used to compute the difference in the rate of AB1 degradation in axenic algal and algal-bacterial consortium treatment setups with varied conditions of iron bioavailability.

| **Parameter** | **Levene** | | **ANOVA** | | | **Tukey** | | | |
| --- | --- | --- | --- | --- | --- | --- | --- | --- | --- |
|  | **Levene Statistic** | ***p* value** | **Sum of squares** | **F statistics** | ***p* value** | **Association of axenic algal w.r.t.** | ***p* value** | **Association of axenic algal (EDTA) w.r.t.** | ***p* value** |
| Rate | 2.784 | 0.110 | 0.03 (Between groups)  0.01  (within groups) | 7.938 | 0.009 | consortium | 0.000 | consortium | 0.000 |
|  |  |  |  |  |  | axenic algal (EDTA) | 0.000 | axenic algal | 0.000 |
|  |  |  |  |  |  | consortium (EDTA) | 0.005 | consortium (EDTA) | 0.126 |

Supplementary Table S5: The L'16 (4^(3)^) orthogonal array design with three factors (Fe conc., pH, and Dye conc.) with their respective levels. The responses were recorded as the rate of degradation (h^-1^) of Acid Black 1 (AB1) dye in the microbial setups with only axenic algal (setup 1) and algal-bacterial consortium (setup 2).

| **Exp.** | **Factors** | | | **Response (Rate, h^-1^)** | | **Degradation (%)** | |
| --- | --- | --- | --- | --- | --- | --- | --- |
|  | **Fe conc.** | **pH** | **Dye conc.** | **axenic algal**  **(setup 1)** | **consortium**  **(setup 2)** | **axenic algal**  **(setup 1)** | **consortium**  **(setup 2)** |
| 1 | 1x10^-7^ | pH6 | 4 µM | 3.69 ± 0.55 x 10^-2^ | 9.10 ± 1.23 x 10^-2^ | 77.36 ± 0.58 | 89.12 ± 1.17 |
| 2 | 1x10^-7^ | pH7 | 8 µM | 5.34 ± 0.19 x 10^-2^ | 6.73 ± 0.09 x 10^-2^ | 81.53 ± 0.75 | 83.04 ± 0.43 |
| 3 | 1x10^-7^ | pH8 | 12 µM | 3.19 ± 0.13 x 10^-2^ | 3.93 ± 0.17 x 10^-2^ | 74.45 ± 1.38 | 81.53 ± 0.44 |
| 4 | 1x10^-7^ | pH9 | 16 µM | 3.39 ± 0.21 x 10^-2^ | 3.38 ± 0.14 x 10^-2^ | 77.17 ± 1.94 | 76.07 ± 0.98 |
| 5 | 1x10^-6^ | pH6 | 8 µM | 3.24 ± 0.08 x 10^-2^ | 4.21 ± 0.45 x 10^-2^ | 76.10± 0.96 | 75.26 ± 1.45 |
| 6 | 1x10^-6^ | pH7 | 4 µM | 3.32 ± 0.21 x 10^-2^ | 5.14 ± 0.56 x 10^-2^ | 80.37 ± 2.34 | 79.78 ± 2.68 |
| 7 | 1x10^-6^ | pH8 | 16 µM | 1.97 ± 0.15 x 10^-2^ | 2.57 ± 0.08 x 10^-2^ | 57.15 ± 4.74 | 67.97 ± 1.22 |
| 8 | 1x10^-6^ | pH9 | 12 µM | 2.17 ± 0.06 x 10^-2^ | 2.66 ± 0.13 x 10^-2^ | 63.01 ± 1.32 | 70.03 ± 2.61 |
| 9 | 2x10^-6^ | pH6 | 12 µM | 1.36 ± 0.11 x 10^-2^ | 1.95 ± 0.12 x 10^-2^ | 49.50 ± 4.16 | 55.64 ± 3.01 |
| 10 | 2x10^-6^ | pH7 | 16 µM | 1.84 ± 0.06 x 10^-2^ | 1.95 ± 0.11 x 10^-2^ | 56.59 ± 1.29 | 54.00 ± 3.65 |
| 11 | 2x10^-6^ | pH8 | 4 µM | 2.08 ± 0.06 x 10^-2^ | 3.56 ± 0.18 x 10^-2^ | 59.26 ± 1.84 | 80.03 ± 3.19 |
| 12 | 2x10^-6^ | pH9 | 8 µM | 1.70 ± 0.08 x 10^-2^ | 2.47 ± 0.18 x 10^-2^ | 50.65 ± 1.04 | 69.06 ± 2.11 |
| 13 | 5x10^-6^ | pH6 | 16 µM | 1.06 ± 0.09 x 10^-2^ | 1.50 ± 0.10 x 10^-2^ | 28.58 ± 7.11 | 50.06 ± 0.57 |
| 14 | 5x10^-6^ | pH7 | 12 µM | 1.67 ± 0.12 x 10^-2^ | 1.48 ± 0.04 x 10^-2^ | 51.73 ± 1.67 | 47.43 ± 2.32 |
| 15 | 5x10^-6^ | pH8 | 8 µM | 1.61 ± 0.14 x 10^-2^ | 2.74 ± 0.21 x 10^-2^ | 51.24 ± 0.73 | 72.19 ± 1.11 |
| 16 | 5x10^-6^ | pH9 | 4 µM | 2.48 ± 0.25 x 10^-2^ | 2.39 ± 0.20 x 10^-2^ | 60.22 ± 2.01 | 68.34 ± 0.56 |

Supplementary Table S6: The output of the multiple linear regression model used to compute the difference in the rate of AB1 degradation in the 16 experiments suggested by Taguchi’s L_16_ (4^3^) orthogonal array in two different setups.

| **Treatments** | **Factors** | **Rank (delta)** | **Multiple linear regression equation** | **R^2^** | **F statistics** | ***p* value** |
| --- | --- | --- | --- | --- | --- | --- |
| axenic algal  (setup 1) | Fe conc. | 1  (0.022) | Rate = 0.0252 + 0.014 (1x10^-7^) + 0.001 (1x10^-6^) – 0.007 (2x10^-6^) | 94.81% | 27.12 | 0.001 |
|  | pH | 3  (0.008) | Rate = 0.0252 - 0.001 (pH6) + 0.005 (pH7) – 0.003 (pH8) |  | 3.2 | 0.105 |
|  | Dye conc. | 2  (0.009) | Rate = 0.0252 + 0.004 (4µM) + 0.004 (8µM) – 0.004 (12µM) |  | 6.25 | 0.028 |
| consortium  (setup 2) | Fe conc. | 1  (0.03) | Rate = 0.0348 + 0.022 (1x10^-7^) + 0.001 (1x10^-6^) – 0.01 (2x10^-6^) | 94.15% | 18.46 | 0.002 |
|  | pH | 3  (0.01) | Rate = 0.0348 - 0.007 (pH6) + 0.003 (pH7) – 0.003 (pH8) |  | 2.78 | 0.133 |
|  | Dye conc. | 2  (0.02) | Rate = 0.0348 + 0.015 (4µM) + 0.005 (8µM) – 0.009 (12µM) |  | 10.92 | 0.008 |

Supplementary Table S7: Estimated model coefficients for means of the 16 experiments suggested by Taguchi’s L_16_ (4^3^) orthogonal array in two different setups.

| **Factors** | **axenic algal (setup 1)** | | | **consortium (setup 2)** | | |
| --- | --- | --- | --- | --- | --- | --- |
|  | **Coefficient** | **t Statistics** | ***p* value** | **Coefficient** | **t Statistics** | ***p* value** |
| Constant | 0.025231 | 24.927 | 0 | 0.034893 | 17.881 | 0 |
| Fe conc. (1x10^-7^) | 0.014302 | 8.158 | **0** | 0.022976 | 6.798 | **0** |
| Fe conc. (1x10^-6^) | 0.001589 | 0.906 | 0.4 | 0.001622 | 0.48 | 0.648 |
| Fe conc. (2x10^-6^) | -0.00773 | -4.409 | **0.005** | -0.01002 | -2.963 | **0.025** |
| pH (pH6) | -0.00136 | -0.776 | 0.467 | 0.007054 | 2.087 | 0.082 |
| pH (pH7) | 0.005244 | 2.991 | **0.024** | 0.003392 | 1.004 | 0.354 |
| pH (pH8) | -0.00305 | -1.737 | 0.133 | -0.00286 | -0.846 | 0.43 |
| Dye conc. (4µM) | 0.004215 | 2.404 | 0.053 | 0.015617 | 4.62 | **0.004** |
| Dye conc. (8µM) | 0.004547 | 2.594 | **0.041** | 0.005527 | 1.635 | 0.153 |
| Dye conc. (12µM) | -0.00422 | -2.409 | 0.053 | -0.0098 | -2.899 | **0.027** |

Supplementary Table S8: The difference in the growth parameters of the microalgae *Chlorella sorokiniana* co-cultured in the presence of siderophore producing bacteria *Ralstonia pickettii* PW2 at different concentrations of iron.

| **Growth parameter** | **1x10^-7^ M** | | **1x10^-6^ M** | | **2x10^-6^ M** | |
| --- | --- | --- | --- | --- | --- | --- |
|  | **axenic algal** | **consortium** | **axenic algal** | **consortium** | **axenic algal** | **consortium** |
| Carrying capacity (k) | 1.11±0.007 x10^7^ | 1.37±0.06 x10^7^ | 4.83±0.22 x10^7^ | 6.28±0.29 x10^7^ | 7.91±0.75 x10^7^ | 7.41±0.17 x10^7^ |
| Growth rate (r) | 4.01±0.15 x10^-2^ | 5.15±0.15 x10^-2^ | 2.92±0.006 x10^-2^ | 4.18±0.37 x10^-2^ | 2.97±0.26 x10^-2^ | 3.58±0.22 x10^-2^ |
| Doubling time (Dt) | 17.32±0.66 | 13.48±0.42 | 23.70±0.05 | 16.82±1.54 | 23.73±2.29 | 19.50±1.17 |
| Area under curve (auc) | 1.86±0.02 x10^9^ | 2.57±0.11 x10^9^ | 6.17±0.28 x10^9^ | 9.68±0.36 x10^9^ | 9.59±0.53 x10^9^ | 8.98±0.48 x10^9^ |

Supplementary Table S9: The output of the linear regression model used to compute the difference in the growth parameters of *Chlorella sorokiniana* co-cultured (with and without bacteria) at the varying concentrations of iron.

| **Growth parameters** | **Fe**  **conc.** | **Linear regression equation** | **R^2^** | **F statistics** | **t  value** | ***p*  value** |
| --- | --- | --- | --- | --- | --- | --- |
| Growth rate (r) | 1x10^-7^ | r = 0.0401 + 0.011 consortium | 87.23% | 27.33 | 5.228 | 0.006 |
|  | 1x10^-6^ | r = 0.029 + 0.012 consortium | 73.82% | 11.28 | 3.358 | 0.028 |
|  | 2x10^-6^ | r = 0.029 + 0.006 consortium | 42.99% | 3.07 | 1.737 | 0.157 |
| Carrying capacity (k) | 1x10^-7^ | k = 1.1x10^7^ + 0.2x10^7^ consortium | 75.17% | 12.11 | 3.48 | 0.025 |
|  | 1x10^-6^ | k = 4.8x10^7^ + 1.4x10^7^ consortium | 79.76% | 15.77 | 3.971 | 0.0165 |
|  | 2x10^-6^ | k = 7.9 x10^7^ – 0.5x10^7^ consortium | 9.53% | 0.42 | -0.642 | 0.551 |
| Area under curve (auc) | 1x10^-7^ | auc = 1.8x10^9^ + 0.7x10^9^ consortium | 90.17% | 36.68 | 6.052 | 0.003 |
|  | 1x10^-6^ | auc = 6.1x10^9^ + 3.5x10^9^ consortium | 93.44% | 56.97 | 7.548 | 0.001 |
|  | 2x10^-6^ | auc = 9.5 x10^9^ - 0.6 x10^9^ consortium | 15.22% | 0.718 | -0.847 | 0.444 |

Supplementary Table S10: The output of the linear regression model used to compute the difference in the ferrireductase activity of *Chlorella sorokiniana* co-cultured (with and without bacteria) at the varying concentrations of iron.

| **Fe conc.** | **Linear regression equation** | **R^2^** | **F statistics** | **t value** | ***p* value** |
| --- | --- | --- | --- | --- | --- |
| 1x10^-7^ | Ferri = 0.015 + 0.009 consortium | 94.70% | 71.51 | 8.456 | 0.001 |
| 1x10^-6^ | Ferri = 0.006 + 0.002 consortium | 89.92% | 35.69 | 5.974 | 0.003 |
| 2x10^-6^ | Ferri = 0.004 – 0.0007 consortium | 19.71% | 0.98 | 1.737 | 0.377 |

Supplementary Table S11: The output of the linear regression model used to compute the difference in the ferrireductase activity of *Chlorella sorokiniana* cultured at the varying concentrations of ferrireductase inhibitor DPI.

| **S.No.** | **DPI conc.** | **Linear regression equation** | **R^2^** | **F statistics** | **t value** | ***p* value** |
| --- | --- | --- | --- | --- | --- | --- |
| 1 | 50µM | Ferri = 0.023 + 0.009 50µM | 98.31% | 155.4 | -18.74 | 0.000 |
| 2 | 100µM | Ferri = 0.023 + 0.002 100µM |  |  | -18.51 | 0.000 |
| 3 | 150µM | Ferri = 0.023 - 0.0007 150µM |  |  | -13.97 | 0.000 |

Supplementary Table S12: The output of Levene’s, One-way ANOVA, and Tukey’s posthoc tests used to compute the difference in the azoreductase activity of *Chlorella sorokiniana* cultured with and without DPI and iron.

| **Assay** | **Levene** | | **ANOVA** | | | **Tukey** | |
| --- | --- | --- | --- | --- | --- | --- | --- |
|  | **Levene Statistic** | ***p* value** | **Sum of squares** | **F statistics** | ***p* value** | **Association wrt DPI-Fe-** | ***p* value** |
| Azoreductase | 0.0482 | 0.704 | 0.000  (Between groups)  0.000  (within groups) | 31.692 | 0.000 | DPI-Fe+ | 0.012 |
|  |  |  |  |  |  | DPI+Fe- | 0.035 |
|  |  |  |  |  |  | DPI+Fe+ | 0.008 |

Supplementary Table S13: The similarities between Ferric reductase and iron transporter proteins in green algae *Chlamydomonas reinhardtii* and *Chlorella sorokiniana*

| **S.No.** | ***Chlamydomonas reinhardtii*** | ***Chlorella sorokiniana* UTEX 1602** | **Similarity** |
| --- | --- | --- | --- |
| 1 | Ferric-chelate reductase/ oxidoreductase (FRE1)  <https://www.ncbi.nlm.nih.gov/protein/122056437> | Ferric-chelate reductase  <https://www.ncbi.nlm.nih.gov/protein/PRW45569.1> | High |
|  |  | Ferric reductase  <https://www.ncbi.nlm.nih.gov/protein/PRW60931.1> | High |
|  |  | Ferric-chelate reductase 1  <https://www.ncbi.nlm.nih.gov/protein/PRW60222.1> | Low |
| 2 | Fe-assimilating protein 1 and 2  <https://www.ncbi.nlm.nih.gov/protein/XP_001693964.1>  <https://www.ncbi.nlm.nih.gov/protein/XP_001693965.1> | Fe-assimilating 1/ 2  <https://www.ncbi.nlm.nih.gov/protein/PRW57721.1/>  <https://www.ncbi.nlm.nih.gov/protein/PRW45734.1/> | Low |
| 3 | Multicopper ferrioxidase (FOX1)  <https://www.ncbi.nlm.nih.gov/protein/XP_001694585.1> | Multicopper ferroxidase  <https://www.ncbi.nlm.nih.gov/protein/PRW39145.1> | High |
| 4 | Iron permease, membrane protein (FTR1)  <https://www.ncbi.nlm.nih.gov/protein/XP_001693245.1> | Calcium-dependent kinase 1-like  <https://www.ncbi.nlm.nih.gov/protein/PRW61083.1> | Low |
| 5 | Iron-nutrition responsive ZIP family transporter (IRT1/2)  <https://www.ncbi.nlm.nih.gov/protein/XP_001693045.1> | Zinc permease family  <https://www.ncbi.nlm.nih.gov/protein/PRW33534.1> | High |

Supplementary Table S14: List of genes and enzymes in the plasma membrane of algae and yeast associated with iron transport. The proteins in *Chlorella sorokiniana* with similar functions as those of *Chlamydomonas reinhardtii* and *Saccharomyces cerevisiae* were identified by using the NCBI BLAST.

| **Species** | **Gene** | **Protein and function** | **Uniprot/NCBI Link** | **Reference** |
| --- | --- | --- | --- | --- |
| **Green Algae-***Chlamydomonas reinhardtii* | *FRE1* | **Ferric-chelate reductase/ oxidoreductase:**  Cell surface iron reductases that reduce Ferric-citrate and siderophore-bound iron | Uniprot:  <https://www.uniprot.org/uniprot/A2I2U7>  NCBI:  <https://www.ncbi.nlm.nih.gov/protein/122056437> | [23, 26] |
|  | *FOX1* | **Multicopper ferrioxidase:**  Plasma membrane-associated multicopper oxidase that reconverts Fe(II) to Fe(III) which is a substrate for associated trivalent cation—specific permease FTR1 | Uniprot:  <https://www.uniprot.org/uniprot/A8IZT9>  NCBI:  <https://www.ncbi.nlm.nih.gov/protein/XP_001694585.1> |  |
|  | *FTR1* | **Iron permease, membrane protein:**  Ferric permease transports Fe(III) into the cell | Uniprot:  <https://www.uniprot.org/uniprot/Q8LL16>  NCBI:  <https://www.ncbi.nlm.nih.gov/protein/XP_001693245.1> |  |
|  | *FEA1/*  *FEA2* | **Fe-assimilating protein 1 and 2:** Responds to iron deficiency in assimilation of iron for FRE1 and FOX1 | Uniprot:  <https://www.uniprot.org/uniprot/Q9LD42>  <https://www.uniprot.org/uniprot/Q38J95>  NCBI:  <https://www.ncbi.nlm.nih.gov/protein/XP_001693964.1>  <https://www.ncbi.nlm.nih.gov/protein/XP_001693965.1> |  |
|  | *IRT1/2* | **Iron-nutrition responsive ZIP family transporter:**  Transportation of divalent metals ions through plasma membrane | Uniprot:  <https://www.uniprot.org/uniprot/A8IW06>  <https://www.uniprot.org/uniprot/A8IW07>  NCBI:  <https://www.ncbi.nlm.nih.gov/protein/XP_001693045.1>  <https://www.ncbi.nlm.nih.gov/protein/XP_001693046.1> |  |
|  | *NRAMP4* | **Natural resistance-associated macrophage protein 4:**  Transportation of divalent metals ions through plasma membrane | NCBI:  <https://www.ncbi.nlm.nih.gov/protein/AAM54030.1> | [26] |
| **Baker’s Yeast-** *Saccharomyces cerevisiae* (strain ATCC 204508 / S288c) | *FRE1/2/3/4* | **Ferric/cupric reductase transmembrane component 1 and 2:**  Cell surface iron reductases that reduce Ferric-citrate and siderophore-bound iron  **Ferric/cupric reductase transmembrane component 3:**  Selectivity for iron in complex with hydroxamate-type siderophore  **Ferric/cupric reductase transmembrane component 4:**  Low-affinity activity on rhodotorulic acid (hydroxamate siderophore) | Uniprot:  <https://www.uniprot.org/uniprot/P32791>  <https://www.uniprot.org/uniprot/P36033>  <https://www.uniprot.org/uniprot/Q08905>  <https://www.uniprot.org/uniprot/P53746>  NCBI:  <https://www.ncbi.nlm.nih.gov/protein/NP_013315.1>  <https://www.ncbi.nlm.nih.gov/protein/NP_012702.1>  <https://www.ncbi.nlm.nih.gov/protein/NP_015026.1>  <https://www.ncbi.nlm.nih.gov/protein/NP_014458.1> | [23, 30, 31] |
|  | *FET3* | **Iron transport multicopper oxidase FET3/ferroxidaseFET3:**  Plasma membrane-associated multicopper oxidase that reconverts Fe(II) to Fe(III) which is a substrate for associated trivalent cation specific Ftr1p | Uniprot:  <https://www.uniprot.org/uniprot/P38993>  NCBI:  <https://www.ncbi.nlm.nih.gov/protein/NP_013774.1> |  |
|  | *FTR1* | **Plasma membrane iron permease/** **high-affinity iron permease FTR1 :**  Ferric permease transports Fe(III) into the cell | Uniprot:  <https://www.uniprot.org/uniprot/P40088>  NCBI:  <https://www.ncbi.nlm.nih.gov/protein/NP_011072.1> |  |
| **Green algae-**  *Chlorella sorokinana* UTEX 1602 | *C2E21_5734* | **Ferric-chelate reductase** | Uniprot:  <https://www.uniprot.org/uniprot/A0A2P6TMJ2>  NCBI:  <https://www.ncbi.nlm.nih.gov/protein/PRW45569.1> | Maybe like FRE1 |
|  | *C2E21_0396* | **Ferric reductase** | Uniprot:  <https://www.uniprot.org/uniprot/A0A2P6U3N8>  NCBI:  <https://www.ncbi.nlm.nih.gov/protein/PRW60931.1> |  |
|  | *C2E21_1040* | **Ferric-chelate reductase 1** | Uniprot: <https://www.uniprot.org/uniprot/A0A2P6U1N0>  NCBI:  <https://www.ncbi.nlm.nih.gov/protein/PRW60222.1> |  |
|  | *C2E21_7043* | **Multicopper ferroxidase** | Uniprot:  <https://www.uniprot.org/uniprot/A0A2P6TIS5>  NCBI:  <https://www.ncbi.nlm.nih.gov/protein/PRW39145.1> | Maybe like FOX1 and FET3 |
|  | *C2E21_0141* | **Calcium-dependent kinase 1-like** | Uniprot:  <https://www.uniprot.org/uniprot/A0A2P6U456>  NCBI:  <https://www.ncbi.nlm.nih.gov/protein/PRW61083.1> | Maybe like FTR1 |
|  | *C2E21_3645/ C2E21_6014* | **Fe-assimilating 1/ 2** | Uniprot:  <https://www.uniprot.org/uniprot/A0A2P6TUJ1>  <https://www.uniprot.org/uniprot/A0A2P6TN26>  NCBI:  <https://www.ncbi.nlm.nih.gov/protein/PRW57721.1/>  <https://www.ncbi.nlm.nih.gov/protein/PRW45734.1/> | Maybe like FEA1/2 |
|  | *C2E21_8904* | **Zinc (Zn2)-Iron (Fe2) Permease (ZIP) Family isoform A, B, and C** | Uniprot:  <https://www.uniprot.org/uniprot/A0A2P6TCZ6>  <https://www.uniprot.org/uniprot/A0A2P6TCZ9>  <https://www.uniprot.org/uniprot/A0A2P6TD01>  NCBI:  <https://www.ncbi.nlm.nih.gov/protein/PRW20517.1>  <https://www.ncbi.nlm.nih.gov/protein/PRW20515.1>  <https://www.ncbi.nlm.nih.gov/protein/PRW20516.1> | Maybe like IRT1/2 |
|  | *C2E21_7513* | **Zinc permease family** | Uniprot:  <https://www.uniprot.org/uniprot/A0A2P6TGW7>  NCBI:  <https://www.ncbi.nlm.nih.gov/protein/PRW33534.1> | Maybe like IRT1/2 |
| **Marine algae-***Phaeodactylum tricornutum* 1055/1 | *ISIP1* | **Iron-starvation induced protein:**  Uptake of siderophore bound iron directly into cell | Uniprot:  <https://www.uniprot.org/uniprot/B7GA90> | [27] |
| **Green alage-***Chlorella vulgaris* | NA | Ferric reductase enzyme activity for iron uptake of hydroxymate siderophore bound iron. | NA | [32] |
| **Marine green algae-** *Chlorococcum littorale* | NA | Ferric reductase enzyme activity  (BPDS-Fe(II) assay) | NA | [33] |
| **Green alage-***Chlorella kessleri* | NA | Ferric chelate reductase enzyme activity (BPDS-Fe(II) assay) | NA | [34] |
| **Marine algae:** *Scrippsiella trochoidea* | NA | Ferric reductase enzyme activity  (BPDS-Fe(II) assay) | NA | [13] |

Supplementary Table S15: The output of Levene’s, One-way ANOVA, and Tukey’s posthoc tests used to compute the difference in the area under curve parameter of *S. plymuthica* and *R. pickettii* grown in the presence of five sugars.

| **Growth parameters** | **Bacteria** | **Levene** | | **ANOVA** | | | **Tukey** | |
| --- | --- | --- | --- | --- | --- | --- | --- | --- |
|  |  | **Levene Statistic** | ***p* value** | **Sum of squares** | **F statistics** | ***p* value** | **Association of growth in Arabinose w.r.t** | ***p* value** |
| area under curve (auc) | *S. plymuthica* PW1 | 1.414 | 0.298 | 0.244 (Between groups)  0.002 (within groups) | 281.236 | 0.000 | Galactose | 0.000 |
|  |  |  |  |  |  |  | Glucose | 0.995 |
|  |  |  |  |  |  |  | Mannose | 1.000 |
|  |  |  |  |  |  |  | Rhamnose | 0.003 |
|  | *R. pickettii* PW2 | 1.439 | 0.291 | 0.911 (Between groups)  0.002 (within groups) | 1.144E3 | 0.000 | Galactose | 0.000 |
|  |  |  |  |  |  |  | Glucose | 0.000 |
|  |  |  |  |  |  |  | Mannose | 0.000 |
|  |  |  |  |  |  |  | Rhamnose | 0.000 |

**Supplementary Table S16:** Output of the PLSP model.

| **Model** | **Original** | **Mean.Boot** | **Std.Error** | **perc.025** | **perc.975** |
| --- | --- | --- | --- | --- | --- |
| Fe -> Axenic | -1.00387 | -1.01305 | 0.557985 | **-2.11357** | **-0.02552** |
| Fe -> Consortium | -1.67153 | -1.72123 | 0.360652 | **-2.58371** | **-1.06323** |
| pH -> Axenic | -0.04825 | -0.0983 | 0.45444 | -1.03041 | 0.704322 |
| pH -> Consortium | -0.86393 | -0.89719 | 0.431874 | **-1.87546** | **-0.03129** |
| Dye -> Axenic | 0.312265 | 0.256248 | 0.502241 | -0.6752 | 1.282918 |
| Dye -> Consortium | -1.27832 | -1.27649 | 0.358985 | **-2.10101** | **-0.56365** |
| inter_Fe.pH -> Axenic | 0.427583 | 0.456992 | 0.483262 | -0.37917 | 1.416964 |
| inter_Fe.pH -> Consortium | 0.732127 | 0.793676 | 0.401672 | **0.153484** | **1.711321** |
| inter_Fe.Dye -> Axenic | -0.27382 | -0.2367 | 0.521685 | -1.25522 | 0.719546 |
| inter_Fe.Dye -> Consortium | 0.909995 | 0.91276 | 0.429781 | **0.146722** | **1.879142** |
| inter_Dye.pH -> Axenic | -0.55408 | -0.48716 | 0.533091 | -1.42219 | 0.630023 |
| inter_Dye.pH -> Consortium | 0.470601 | 0.480495 | 0.421655 | -0.33954 | 1.422557 |

**5. Supplementary References**

1. Schwyn B, Neilands JB. Universal chemical assay for the detection and determination of siderophores. *Anal Biochem* 1987; **160**: 47–56.

2. Khilyas I V., Shirshikova T V., Matrosova LE, Sorokina A V., Sharipova MR, Bogomolnaya LM. Production of siderophores by *Serratia marcescens* and the role of MacAB efflux pump in siderophores secretion. *Bionanoscience* 2016; **6**: 480–482.

3. Wongsa P, Tanaka M, Ueno A, Hasanuzzaman M, Yumoto I, Okuyama H. Isolation and characterization of novel strains of *Pseudomonas aeruginosa* and *Serratia marcescens* possessing high efficiency to degrade gasoline, kerosene, diesel oil, and lubricating oil. *Curr Microbiol* 2004; **49**: 415–422.

4. Bhatt G, Denny TP. *Ralstonia solanacearum* iron scavenging by the siderophore staphyloferrin B is controlled by PhcA, the global virulence regulator. *J Bacteriol* 2004; **186**: 7896–7904.

5. Sun J, Hu Y, Li W, Zhang Y, Chen J, Deng F. Sequential decolorization of azo dye and mineralization of decolorization liquid coupled with bioelectricity generation using a pH self-neutralized photobioelectrochemical system operated with polarity reversion. *J Hazard Mater* 2015; **289**: 108–117.

6. Tang X, He LY, Tao XQ, Dang Z, Guo CL, Lu GN, et al. Construction of an artificial microalgal-bacterial consortium that efficiently degrades crude oil. *J Hazard Mater* 2010; **181**: 1158–1162.

7. Ramanan R, Kim BH, Cho DH, Oh HM, Kim HS. Algae-bacteria interactions: Evolution, ecology and emerging applications. *Biotechnol Adv* 2016; **34**: 14–29.

8. Baglieri A, Sidella S, Barone V, Fragalà F, Silkina A, Nègre M, et al. Cultivating *Chlorella vulgaris* and *Scenedesmus quadricauda* microalgae to degrade inorganic compounds and pesticides in water. *Environ Sci Pollut Res* 2016; **23**: 18165–18174.

9. Sprouffske K, Wagner A. Growthcurver: An R package for obtaining interpretable metrics from microbial growth curves. *BMC Bioinformatics* 2016; **17**: 172.

10. Wan M, Jin X, Xia J, Rosenberg JN, Yu G, Nie Z, et al. The effect of iron on growth, lipid accumulation, and gene expression profile of the freshwater microalga *Chlorella sorokiniana*. *Appl Microbiol Biotechnol* 2014; **98**: 9473–9481.

11. Keshtacher-Liebson E, Hadar Y, Chen Y. Oligotrophic bacteria enhance algal growth under iron-deficient conditions. *Appl Environ Microbiol* 1995; **61**: 2439–2441.

12. Kurth C, Wasmuth I, Wichard T, Pohnert G, Nett M. Algae induce siderophore biosynthesis in the freshwater bacterium Cupriavidus necator H16. *BioMetals* 2019; **32**: 77–88.

13. Amin SA, Green DH, Hart MC, Kupper FC, Sunda WG, Carrano CJ, et al. Photolysis of iron-siderophore chelates promotes bacterial-algal mutualism. *Proc Natl Acad Sci* 2009; **106**: 17071–17076.

14. Rajapitamahuni S, Bachani P, Sardar RK, Mishra S. Co-cultivation of siderophore-producing bacteria *Idiomarina loihiensis* RS14 with *Chlorella variabilis* ATCC 12198, evaluation of micro-algal growth, lipid, and protein content under iron starvation. *J Appl Phycol* 2019; **31**: 29–39.

15. Tanoi T, Kawachi M, Watanabe MM. Effects of carbon source on growth and morphology of *Botryococcus braunii*. *J Appl Phycol* 2011; **23**: 25–33.

16. Mühlenbruch M, Grossart HP, Eigemann F, Voss M. Mini-review: Phytoplankton-derived polysaccharides in the marine environment and their interactions with heterotrophic bacteria. *Environ Microbiol* 2018; **20**: 2671–2685.

17. Watanabe K, Takihana N, Aoyagi H, Hanada S, Watanabe Y, Ohmura N, et al. Symbiotic association in *Chlorella* culture. *FEMS Microbiol Ecol* 2005; **51**: 187–196.

18. Sambles C, Moore K, Lux TM, Jones K, Littlejohn GR, Gouveia JD, et al. Metagenomic analysis of the complex microbial consortium associated with cultures of the oil-rich alga Botryococcus braunii. *Microbiologyopen* 2017; **6**: e00482.

19. Kamalanathan M, Doyle SM, Xu C, Achberger AM, Wade TL, Schwehr K, et al. Exoenzymes as a Signature of Microbial Response to Marine Environmental Conditions. *mSystems* 2020; **5**.

20. Seymour JR, Amin SA, Raina JB, Stocker R. Zooming in on the phycosphere: The ecological interface for phytoplankton-bacteria relationships. *Nat Microbiol* 2017; **2**: 17065.

21. Sutak R, Camadro JM, Lesuisse E. Iron Uptake Mechanisms in Marine Phytoplankton. *Front Microbiol* 2020; **11**: 2831.

22. Coale TH, Moosburner M, Horák A, Oborník M, Barbeau KA, Allen AE. Reduction-dependent siderophore assimilation in a model pennate diatom. *Proc Natl Acad Sci U S A* 2019; **116**: 23609–23617.

23. Allen MD, Del Campo JA, Kropat J, Merchant SS. FEA1 , FEA2 , and FRE1 , Encoding Two Homologous Secreted Proteins and a Candidate Ferrireductase, Are Expressed Coordinately with FOX1 and FTR1 in Iron-Deficient Chlamydomonas reinhardtii. *Eukaryot Cell* 2007; **6**: 1841–1852.

24. Amin SA. The role of siderophores in algal-bacterial interactions in the marine environment. 2010. UC San Diego.

25. Lelandais G, Scheiber I, Paz-Yepes J, Lozano JC, Botebol H, Pilátová J, et al. Ostreococcus tauri is a new model green alga for studying iron metabolism in eukaryotic phytoplankton. *BMC Genomics* 2016; **17**: 1–23.

26. Blaby-Haas CE, Merchant SS. The ins and outs of algal metal transport. *Biochim Biophys Acta - Mol Cell Res* 2012; **1823**: 1531–1552.

27. Kazamia E, Sutak R, Paz-Yepes J, Dorrell RG, Vieira FRJ, Mach J, et al. Endocytosis-mediated siderophore uptake as a strategy for Fe acquisition in diatoms. *Sci Adv* 2018; **4**: eaar4536.

28. Sutak R, Botebol H, Blaiseau P-L, Léger T, Bouget F-Y, Camadro J-M, et al. A comparative study of iron uptake mechanisms in marine microalgae: Iron binding at the cell surface is a critical step. *Plant Physiol* 2012; **160**: 2271–2284.

29. Sutak R, Šlapeta J, Roman MS, Camadro JM, Lesuisse E. Nonreductive iron uptake mechanism in the marine alveolate *Chromera velia*. *Plant Physiol* 2010; **154**: 991–1000.

30. Dancis A, Roman DG, Anderson GJ, Hinnebusch AG, Klausner RD. Ferric reductase of Saccharomyces cerevisiae: molecular characterization, role in iron uptake, and transcriptional control by iron. *Proc Natl Acad Sci U S A* 1992; **89**: 3869–73.

31. Dancis A, Klausner RD, Hinnebusch AG, Barriocanal JG. Genetic evidence that ferric reductase is required for iron uptake in Saccharomyces cerevisiae. *Mol Cell Biol* 1990; **10**: 2294–2301.

32. Allnutt FCT, Bonner WD. Characterization of Iron Uptake from Ferrioxamine B by Chlorella vulgaris. *Plant Physiol* 1987; **85**: 746–750.

33. Sasaki T, Kurano N, Miyachi S. Induction of Ferric Reductase Activity and of Iron Uptake Capacity in. *Plant Cell Physiol* 1998; **39**: 405–410.

34. Weger HG, Middlemiss JK, Petterson CD. Ferric chelate reductase activity as affected by the iron-limited growth rate in four species of unicellular green algae (Chlorophyta). *J Phycol* 2002; **38**: 513–519.
